# Modular design of *Salmonella* Typhimurium OMV-based nanovaccines for tunable dual-form polysaccharide antigen delivery

**DOI:** 10.1371/journal.ppat.1014781

**Published:** 2026-11-04

**Authors:** Mengru Li, Kai Zhou, Meihong Lei, Huanhuan Cao, Xinyu Liu, Shan Li, Ruida Wu, Ming Yang, Xiaoping Bian, Longlong Cao, Qingke Kong

**Affiliations:** 1 College of Veterinary Medicine, Southwest University, Beibei, Chongqing, China; 2 College of Veterinary Medicine, Shandong Agricultural University, Taian, Shandong, China; Universite Paris Descartes Faculte de Medecine, FRANCE

## Abstract

Bacterial diseases are primarily managed with antibiotics, but vaccination is widely acknowledged as the most efficacious strategy for their prevention. Bacterial polysaccharides serve as key protective antigens in hosts and are therefore consistently prioritized as targets for bacterial vaccine development. Polysaccharide-conjugated vaccines have emerged as a promising and effective approach to combat multidrug-resistant bacteria. In order to construct a vaccine carrier capable of delivering bacterial polysaccharides while inducing robust immune response, this study utilized outer membrane vesicles (OMVs) derived from *Salmonella* Typhimurium as the vaccine carrier instead of the traditional adjuvant components to enhance the immunogenicity of polysaccharide vaccine. *S.* Typhimurium was genetically engineered to enable the controlled synthesis of LPS and glycoprotein, which were encapsulated within OMVs as two distinct polysaccharide vaccine formulations. This engineered delivery system was used to deliver the O-antigen polysaccharide of *Shigella flexneri* 3a, and subsequent validation in a murine model further confirmed that its ability to elicit an effective in vivo immune response. The polysaccharide delivery carrier platform established in this study provides a foundation for the subsequent multi-form delivery of bacterial polysaccharides in next-generation biological vaccines.

## Author summary

The vaccine platform can standardize the induction of immune responses in the body with high safety and low adverse reactions. Its design also allows for the easy switching of the displayed antigens, thereby achieving high efficacy and wide adaptability. The mutant strain of *Salmonella* Typhimurium constructed in this study can successfully control the synthesis of O antigen polysaccharide LPS and glycoproteins by adding inducers. This enables the O antigen polysaccharide of the bacteria to serve as a polysaccharide substrate for the synthesis of both LPS and glycoproteins, achieving the purpose of artificially intervening in the delivery form of OMVs. Moreover, by replacing the expression plasmids of polysaccharide antigens and peptide antigens, this vaccine platform can achieve adaptability and universality for different bacterial antigens, providing a foundation for the development of vaccines for different bacteria. Finally, through animal experiments, it has been confirmed that the controllable OMVs-based polysaccharide vaccine delivery platform constructed in this study can effectively induce the production of specific antibody levels in the body, thereby exerting immune protection effects, and at the same time, it has excellent biological safety.

## Introduction

Advancements in nanotechnology have propelled nanoparticle-based nanovaccines to the forefront of vaccinology research. The research and development of nanovaccines can be primarily categorized into two main types [1]. The first category involves synthetic nanomaterials serving as delivery carriers, including liposomes, gold nanoparticles, polymeric nanoparticles, carbon nanotubes, and other synthetic nanoparticles [2–5], and the second category encompasses natural nanoscale particles, such as virus-like particles (VLPs), bacterial ghosts, minicells, and membrane vesicles (MVs) [6–8].

Bacterial Outer membrane vesicles (OMVs) are vesicle-like structures naturally secreted by Gram-negative bacteria during growth. They are bilayer-membrane spherical particles with an average diameter of 20–200 nm, composed primarily of lipopolysaccharides, phospholipids, a small amount of peptidoglycan, and proteins localized in periplasm and bacterial outer membrane [9,10]. Most proteins enriched in OMVs are virulence factors, such as enterotoxins, hemolysin, and flagellin, which mediate host cell specific targeting, host cell damage, regulation of host immune response, transfer of antibiotic resistance, and coordinated secretion with other bacterial effectors, biofilm formation and intercellular communication [11–13]. OMVs possess a high capacity to induce polysaccharide antigen-specific immune responses, making them idea carrier delivering low immunogenicity antigens such as polysaccharide (PS) or peptides [14]. Additionally, OMVs can serve as carriers delivering chemically or genetically linked polysaccharides, enabling the direct exposure of polysaccharides or proteins on the vesicles, such as polysaccharide antigens from *Neisseria meningitidis* serogroups A and C, *Haemophilus influenza* type b, *Streptococcus* group A, and *Salmonella* Typhi Vi [15–18].

OMVs offer unique advantages as a nano-vaccine platforms: (1) OMVs possess intrinsic adjuvant properties, with surface components recognized by the host as pathogen-associated molecular patterns (PAMPs) [19]. (2) The nanostructure of OMVs facilitates direct entry into the lymphatic system and enhances presentation by antigen-presenting cells (APCs), thereby enhancing immune stimulation. (3) OMVs are non-replicating nanoparticles, eliminating the need for inactivating agents’ post-purification, which helps maintain the natural state of the antigen. (4) The purification process is relatively straightforward, achievable via ultra-centrifugation of the bacterial culture supernatant. (5) Antigens can be incorporated into either the outer membrane or the inner cavity of OMVs, allowing for the simultaneous expression of multiple antigens. (6) OMVs exhibit high stability, including at room temperature, making OMV-based vaccines easier to store and transport compared to mRNA vaccines [20,21]. Through genetic engineering modification, *Salmonella* OMVs can carry different types of heterologous antigens such as proteins, polysaccharides or nucleic acids, and display them on the surface of the OMVs or load them in the inner cavity of the vesicles. At the same time, they can induce the body to produce specific antibodies against the *Salmonella* itself and the heterologous antigens, providing the body with the ability to resist Salmonella and the corresponding pathogen infections.

A major challenge in LPS/O-PS-based vaccine development is the poor immunogenicity of purified polysaccharides in infants and children, despite moderately immunogenic in adults. Since polysaccharide antigens are classified as T-cell-independent antigens, which can only stimulate B cells to produce low-affinity IgM antibodies [22]. Research has indicated that bacterial polysaccharide antigens, such as O-polysaccharide (OPS) or capsular polysaccharide (CPS), can be covalently linked to suitable carrier proteins to form glycoproteins [23]. These glycoproteins act as T cell-dependent antigens, capable of activating T cells and inducing B cells to produce specific high-affinity IgG, IgA, and other antibodies, while also generating memory cells [24,25]. In the late 1980s and early 1990s, a glycoconjugate vaccine for influenza type b was licensed in industrialized countries, including the United Kingdom and the United States. This vaccine has since been utilized for the routine immunization of infants [26]. Subsequently, commercially polysaccharide conjugate vaccines targeting *Haemophilus influenzae*, *Streptococcus pneumoniae*, and *N. meningitidis* were successfully developed, demonstrating high immunogenicity and safety [27,28].

Traditional chemical cross-linking for glycoconjugate vaccine production suffers from cumbersome preparation process, high production costs, and an uncontrollable reaction conditions, which complicate standardization efforts [29,30]. Thus, bio-enzymatic synthesis of glycoproteins has emerged as a focus of current bacterial vaccine development. Bacterial oligosaccharyltransferases (OTases) are generally classified into two categories: N-linked OTases, which catalyze the covalent bonding of preassembled oligosaccharides to the side chains of asparagine residues in proteins, and O-linked OTases, which typically transfer polysaccharides to the side chains of serine or threonine residues [31]. Both types are membrane-bound enzymes located in the inner membrane, glycosylating proteins in the periplasmic space with glycans derived from lipid-linked precursors. Currently, two types of OTases were widely utilized for the synthesis of glyco-conjugates include the *Campylobacter jejuni* N-OTase PglB and the *N. meningitidis* O-OTase PglL, both of which demonstrate significant promiscuity toward glycan substrates [32–34]. PglB requires the presence of an acetamido group at the C2 position of the reducing-end monosaccharide of the glycan substrate and no β1–4 glycosidic bond between the first two monosaccharides. In contrast, PglL exhibits a broader substrate specificity for glycan chains but is unable to transfer reducing-end monosaccharides to glucose polysaccharides. Additionally, other O-linked OTases have been identified, including PglS, TfpO, and TfpM [35].

OMVs are increasingly utilized as vaccine carriers to deliver various antigens in a range of clinical vaccine research studies. We hypothesis that co-delivery of two distinct polysaccharide formats by OMVs may induce unexpected immune responses and enhance protective efficacy. In this study, we describe a nanovaccine platform using *S.* Typhimurium OMVs for co-delivery of bacterial LPS and glycoproteins. We selected a *S.* Typhimurium mutant lacking the *rfbP* gene, which precludes the synthesis of *Salmonella* O-antigen polysaccharides, and introduced exogenous plasmids for heterologous polysaccharides synthesis. The *waaL* gene encoding the glycosyltransferase WaaL was regulated by an arabinose-inducible promoter to control LPS synthesis. Furthermore, the P_trc_ promoter was used to regulate expression of the carrier protein cholera toxin B subunit (CTB) fused with the PilE^S45-K73^ fragment and the heteroglycosyltransferase PglL expression for glycoprotein synthesis, and controlled synthesis of LPS and glycoprotein were achieved as two distinct polysaccharide vaccine formulations, and encapsulated within OMVs. Finally, we assessed the immunogenic effect of the vaccine using *Shigella flexneri* 3a polysaccharide as a model antigen.

## Results

### Construction of *Salmonella* Typhimurium OMVs for internal delivery of bacterial O-antigen polysaccharides

The laboratory-preserved *S.* Typhimurium ST1 strain (ST ∆*ompACD* ∆*fljB* ∆*fliC* ∆*rfbP*) was used as the parental strain. Deletion of *rfbP* abrogates endogenous O-antigen synthesis, while deletion of flagellar (*fljB*, *fliC*) and several outer membrane proteins (*ompACD*) genes enhances OMV purity. A recombinant expression plasmid containing signal peptide of OmpD was constructed to localize the model carrier protein CTB to the periplasmic side of the bacterial outer membrane. Heterologous glycosyltransferase was introduced to achieve protein glycosylation, thereby establishing an OMVs carrier platform for the internal delivery of bacterial O-antigen polysaccharides (Fig 1A).

**Fig 1:**
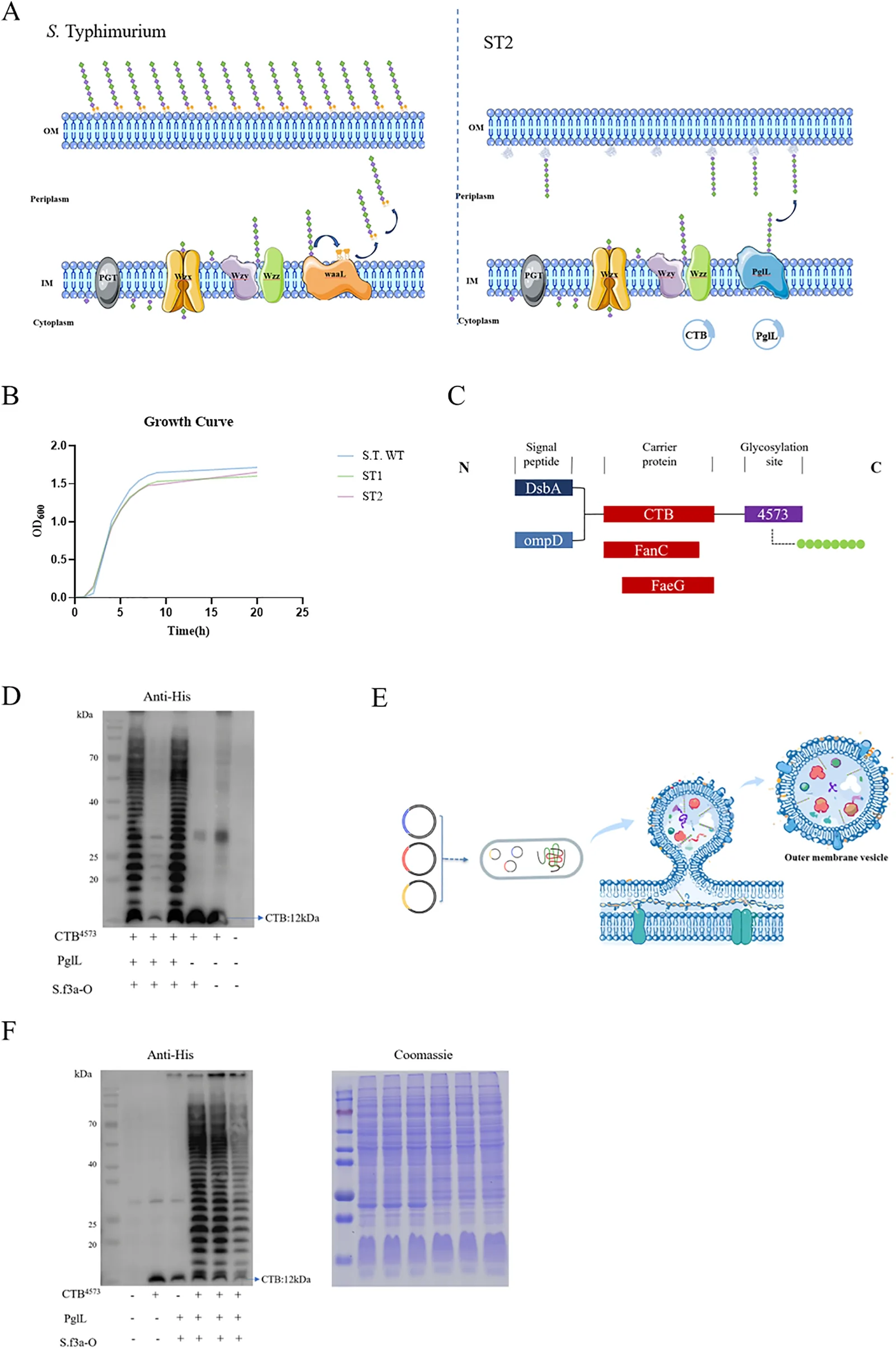
Construction and validation of an OMV-based platform for internal delivery of glycoproteins. **(a)** Schematic diagram depicting the pattern of glycoproteins displayed on the periplasmic side of the cell membrane. **(b)** Growth curve of bacterial strain ST2 (ST ∆*ompACD* ∆*fljB* ∆*fliC* ∆*rfbP* ∆*waaL*) lacking the glycosyltransferase WaaL. **(c)** Modular design scheme of carrier protein recombinant plasmid. **(d)** Detection of *S. flexneri* 3a glycoprotein expression in strain ST2. Herein, the signal peptide of the carrier protein is the DsbA signal peptide. **(e)** Schematic diagram of the internal delivery mode of glycoproteins in *Salmonella* Typhimurium OMVs. **(f)** Detection of *S. flexneri* 3a glycoprotein expression in strain ST2. Herein, the signal peptide of the carrier protein is the OmpD signal peptide.

First, the *waaL* deletion strain ST2 was successfully generated from ST1 via suicide plasmid-mediated homologous recombination (S1 Fig). ST2 exhibited a slightly slower growth rate compared to the parent strain ST1 (Fig 1B). The plasmid responsible for synthesis of O-antigen polysaccharide of *S. flexneri* serotype 3a was electroporated into ST2, and silver staining revealed that the corresponding LPS was not synthesized (S2 Fig). After confirming that ST2 could not synthesize LPS, two plasmids encoding the carrier protein CTB (pMAL-1) and the glycosyltransferase PglL (pACT3-1) were electroporated into ST2. In ST2, PglL successfully catalyzed the conjugation of exogenous O-antigen polysaccharide of *S. flexneri* serotypes 2a, 3a, and 6 in the periplasmic space onto the carrier protein CTB, which contains the DsbA signal peptide and glycosylation sites (Fig 1C, S3 Fig). Next, the plasmids for synthesizing the CTB carrier protein (pMAL-2) containing signal peptide of OmpD and PglL, and for synthesizing O-antigen polysaccharides of *S. flexneri* 2a, 3a, and 6 (pYA3337-1, pYA3337-2, pYA3337-3) were co-transformed into ST2 to verify glycoprotein production (Fig 1D and S4 Fig). Using His-tag antibodies, SDS-PAGE and Western blot experiments confirmed that the carrier protein successfully underwent glycosylation following IPTG induction (Fig 1F, S5 Fig). OMVs were extracted from the supernatant of the culture medium via ultracentrifugation, and Western blot confirmed that O-antigen polysaccharides were successfully delivered in the form of glycoproteins within OMVs (S6 Fig).

### Construction of *S.* Typhimurium OMVs platform for simultaneous internal and external delivery of bacterial polysaccharides

We have demonstrated that the carrier protein CTB can be localized to the periplasmic side of the outer membrane of *S.* Typhimurium via the OmpD signal peptide, and that introduction of PglL-encoding plasmid pACT3–1 into ST2 enables conjugation of *Shigella* O-antigen polysaccharides to CTB. We further explore whether it is possible to introduce the heterologous glycosyltransferase PglL while retaining the endogenous glycosyltransferase WaaL, thus enabling simultaneous delivery of polysaccharides both internally as glycoproteins within the OMVs and externally as LPS to improve the immunity. First, CTB expression plasmid containing DsbA signal peptide and the PglL expression plasmid were co-transformed into *S. flexneri* 3a wild-type. Western blot and silver staining confirmed that the O-antigen polysaccharide could be successfully synthesized into LPS and glycosylated onto the carrier protein to form a glycoprotein; however, glycoprotein synthesis in wild-type bacteria was relatively less efficient than in ST1, likely due to the preferential synthesis of endogenous LPS, thereby presumably exerting a suppressive effect on glycoprotein biosynthesis (Fig 2A). To achieve internal delivery of glycoproteins via OMVs, CTB was localized to the periplasmic side of the outer membrane using the OmpD signal peptide. This localization of the carrier protein to the outer membrane resulted in significantly higher glycosylation in comparison with its periplasmic localization (Fig 2B). Meanwhile, parallel experiments with wild-type *S.* Typhimurium yielded the similar result as that obtained in *Shigella* (Fig 2C, 2D). To explore the feasibility of OMVs-mediated on-demand delivery of diverse polysaccharides, heterologous O-antigen polysaccharides were synthesized in strain ST1, and western blot and silver staining demonstrated that the O-antigen polysaccharide of *S. flexneri* 3a coexisted in strain ST1 as both LPS and glycoprotein forms (Fig 2E). Our results further revealed that the glycosylation efficiency in the WaaL-competent group was slightly lower than that in the WaaL-deficient group, indicating that concurrent synthesis of LPS and glycoproteins affects the yield of glycoproteins.

**Fig 2:**
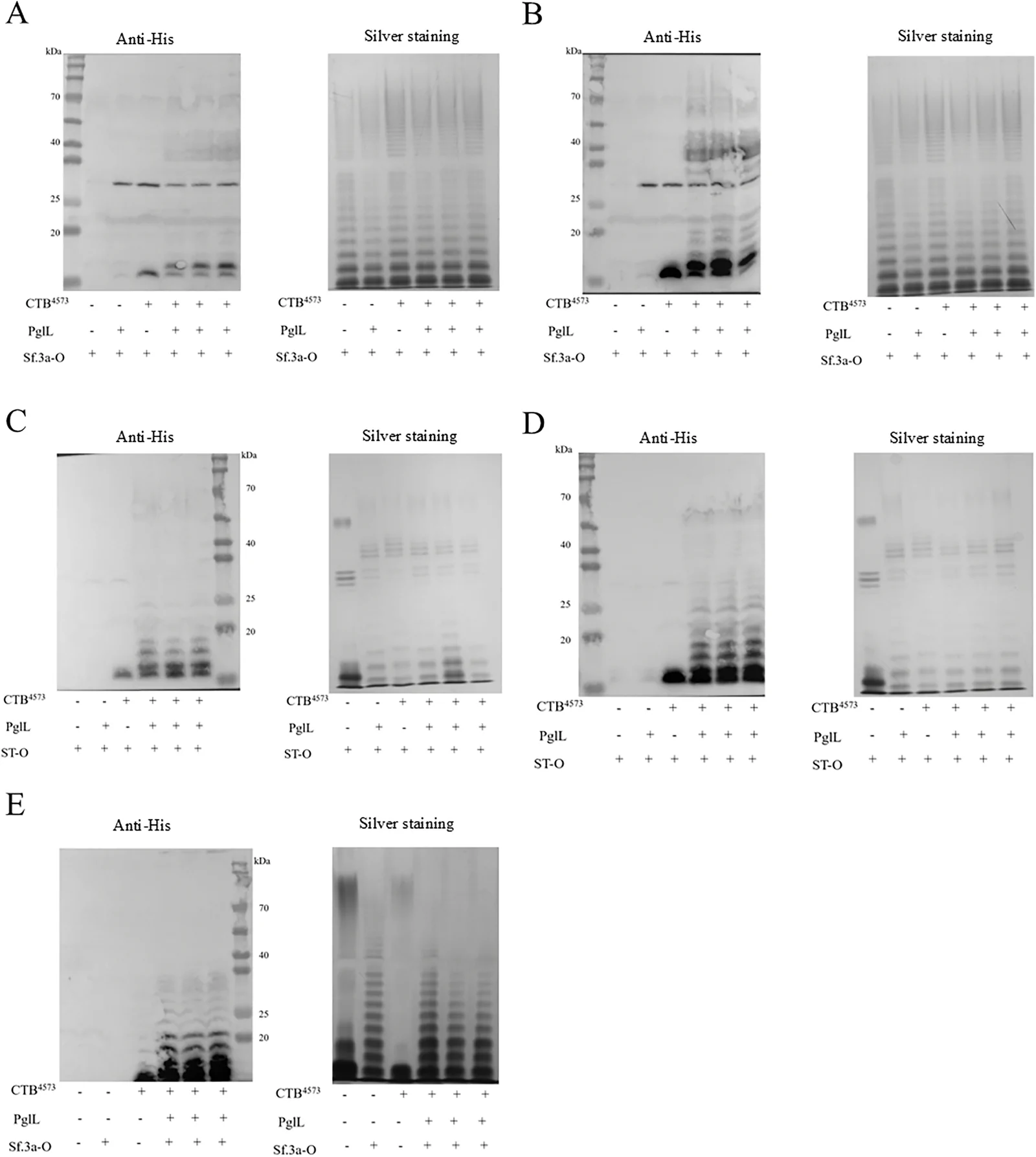
Verification of the coexistence of LPS and glycoproteins within bacteria. **(a)** Verification of the simultaneous formation of glycoproteins in the periplasmic space and LPS outside the outer membrane in wild-type *S. flexneri* 3a. CTB was located in the periplasmic space. **(b)** Verification of the simultaneous formation of glycoproteins on the periplasmic side of the outer membrane and LPS outside the outer membrane in wild-type *S. flexneri* 3a. CTB is located in the outer membrane. **(c)** Verification of the simultaneous formation of glycoproteins in the periplasmic space and LPS outside the outer membrane in wild-type ***S.*** Typhimurium. CTB was located in the periplasmic space **(d)** Verification of the simultaneous formation of glycoproteins on the periplasmic side of the outer membrane and LPS outside the outer membrane in wild-type ***S.*** Typhimurium. CTB is located in the outer membrane. **(e)** Verification of the O-antigen polysaccharide of *Shigella flexneri* 3a forming glycoproteins on the periplasmic side of the outer membrane and LPS on the outer membrane surface in wild-type ***S.*** Typhimurium.

### Construction of an OMVs platform capable of regulating internal and external delivery of O-antigen polysaccharides of *S.* Typhimurium

To establish an OMVs delivery platform capable of on-demand production of glycoproteins and/or LPS, we genetically modified the promoter of the glycosyltransferase encoding gene *waaL*, thereby achieving arabinose-regulated LPS biosynthesis (Fig 3A). Initially, homologous recombination mediated by a suicide plasmid was employed to delete the *pagL* gene in ST2 strain, leading to the generation of the mutant strain designated ST3. Subsequently, the *waaL* gene, under the transcriptional control of the arabinose-inducible *araC* P_BAD_ promoter, was genetically integrated into the original *pagL* locus to regulate LPS synthesis, yielding strain ST4 (S7 Fig).

**Fig 3:**
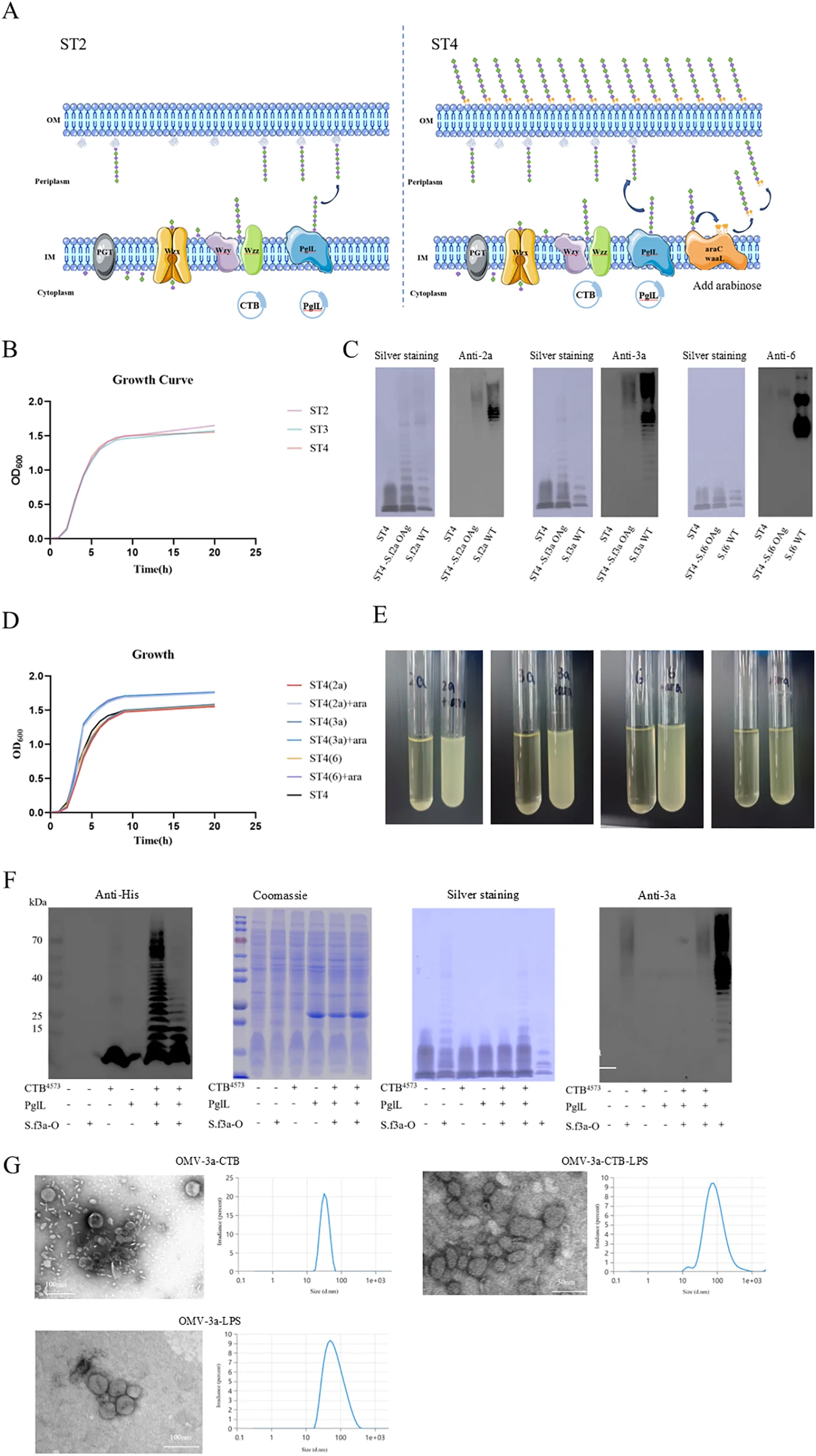
Construction and validation of the OMVs platform for internal delivery of glycoproteins and external controlled delivery of LPS. **(a)** Schematic diagram of the OMVs platform for controllable external delivery of LPS and internal delivery of glycoproteins. **(b)** Growth curve of the bacterial strain ST4(ST ∆*ompACD* ∆*fljB* ∆*fliC* ∆*rfbP* ∆*waaL* ∆*pagL*::*araC*P_BAD_ *waaL*) containing the *waaL* gene with an arabinose promoter inserted at the *pagL* gene locus. **(c)** Characterization of strain ST4(pYA3337-1), ST4(pYA3337-2), ST4(pYA3337-3) using anti-*S. flexneri* serotypes 2a, 3a, and 6 antibodies via silver staining and Western blot. **(d)** Growth curve of the bacterial strain ST4 (pYA3337-1), ST4 (pYA3337-2), ST4 (pYA3337-3) with and without arabinose supplementation. **(e)** Observation of bacterial oscillatory growth patterns in ST4 (pYA3337-1), ST4 (pYA3337-2), ST4 (pYA3337-3) bacteria with and without arabinose supplementation. **(f)** Verification of glycoprotein and LPS synthesis in strain ST4 (pYA3337-2) with and without arabinose using silver staining and Western Blot analysis with anti-His and anti-*S. flexneri* serotype 3a sera. **(g)** TEM images of 3a-CTB, OMV-3a-CTB, OMV-3a-LPS. Bar = 100 nm.

Bacterial growth curve showed no significant difference between ST3, ST4 and the parental strain ST2 (Fig 3B). The large plasmids carrying the O-antigen polysaccharide genes of *S. flexneri* serotypes 2a, 3a, and 6 were introduced into ST4 via electroporation, and Western blot and silver staining confirmed that LPS was successfully synthesized only in the arabinose-supplemented groups (Fig 3C).

Preliminary studies revealed that bacteria lacking LPS synthesis exhibited a higher sedimentation coefficient and slower growth rates. Observations of bacterial shaking culture revealed that ST4 strains synthesizing heterologous O-antigen polysaccharides under arabinose supplementation maintained a suspended growth state and exhibited relatively rapid growth rates, overcoming the sedimentation and growth defects associated with LPS deficiency (Fig 3D, 3E). Collectively, these results demonstrated that the arabinose-inducible WaaL enabled controllable LPS biosynthesis in ST4.

Recombinant plasmid containing carrier protein CTB with OmpD signal peptide and PglL was electroporated into ST4, which harbors large plasmids for synthesizing O-antigen polysaccharide of *S. flexneri* serotypes 2a, 3a, 6. To confirm whether the modified strain could simultaneously synthesize the heterologous LPS and glycoprotein under the regulation of arabinose, SDS-PAGE and Western blot using His-Tag antibodies confirmed that glycoproteins were successfully synthesized under PglL catalysis regardless of arabinose induction (Fig 3F, S8 Fig). Western blot and silver staining using bacterial-specific serum antibodies demonstrated that bacteria supplemented with arabinose were capable of synthesizing the corresponding LPS meanwhile producing glycoproteins. Furthermore, after quantification of the extracted LPS, the results showed that the synthesis amount of LPS in the arabinose-dependent group was lower than that in the normal synthesis group. This result indirectly proves that when glycoproteins and lipopolysaccharides are synthesized together, ST4 is more suitable as a polysaccharide synthesis substrate compared to ST1 (S1 Table). Transmission electron microscopy (TEM) revealed distinct OMVs morphologies based on their different polysaccharide delivery modes: LPS-decorated OMVs exhibited a prominent electron-dense outer layer, while LPS-deficient OMVs appeared transparent (Fig 3G).

### Application of diverse heterologous polysaccharides and carrier proteins in the OMVs platform

To validate this platform versatility, two additional carrier proteins including *E. coli* fimbrial proteins FanC and FaeG were tested alongside a widely used model carrier protein CTB. Recombinant plasmids encoding the FanC and FaeG carrier proteins with the N-terminal linked to OmpD signal peptide and the C-terminal fused to the glycosylation site 4573 were electroporated into ST2. Western blot confirmed that both proteins were expressed in ST2 with expected molecular weights (Fig 4A). Next, *S. flexneri* 3a O-antigen and PglL plasmids were transformed into ST2. The results confirmed that PglL catalyzed the glycosylation of *S*. *flexneri* 3a O-antigen polysaccharides onto the carrier proteins with localization to the periplasmic side of outer membrane (Fig 4B, 4C and S9 Fig). After plasmid transformation into ST1, Western blot and silver staining verified simultaneous production of glycoprotein and LPS in the presence of FanC or FaeG with higher glycoprotein synthesis in ST2 than in ST1, yielding results similar to CTB (Fig 4D, 4E). In ST4, arabinose induction (0.05%–0.2%) did not significantly affect glycoprotein/LPS yields, while the absence of arabinose reduced bacterial growth and glycoprotein production (Fig 4F, 4G).

**Fig 4:**
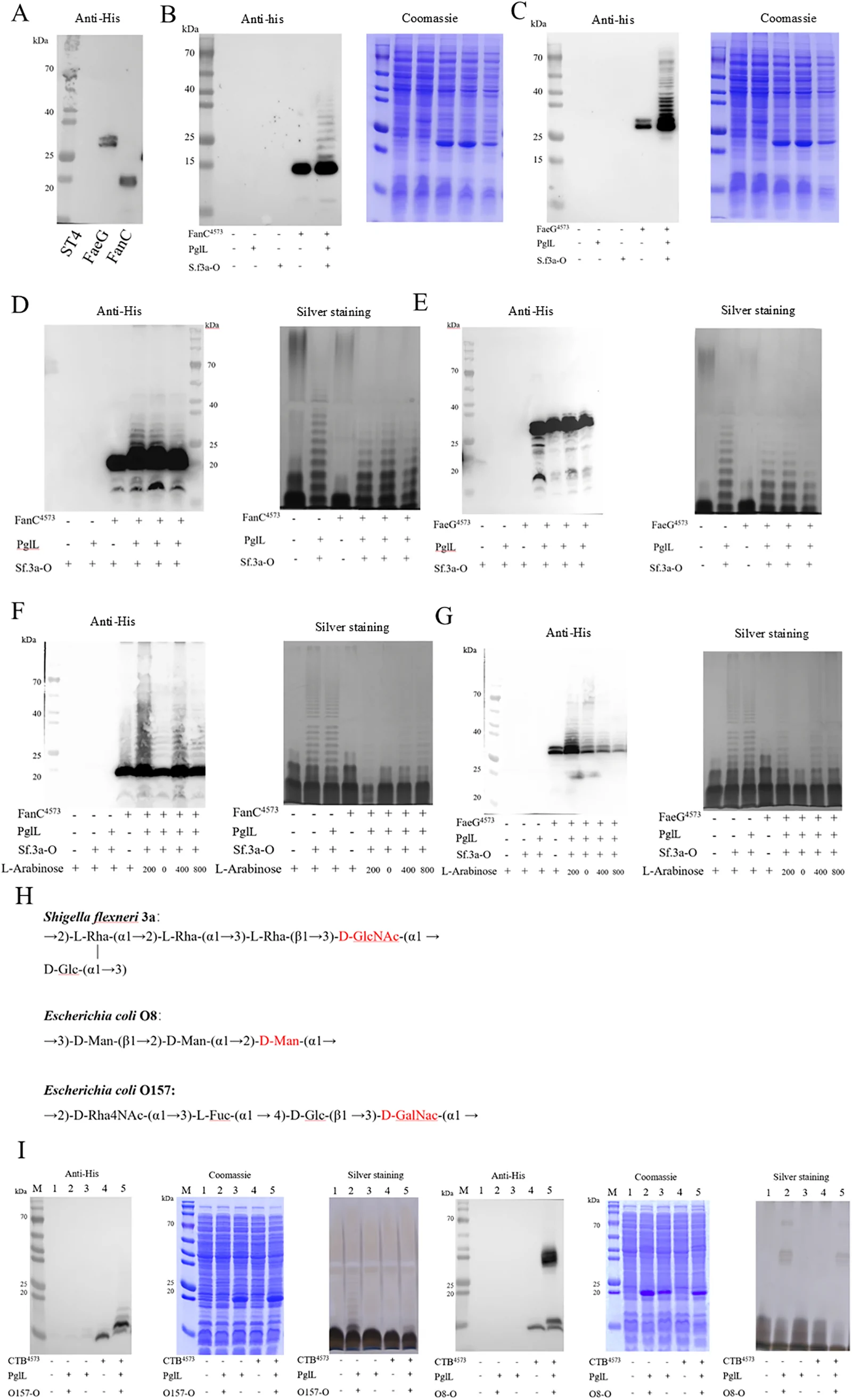
Utilization of diverse carrier proteins and polysaccharides in an OMV-based delivery platform. **(a)** Detection of acceptor protein FanC and FaeG expression in strain ST4. **(b)** Validation of glycosylation of carrier proteins FanC with *S. flexneri* 3a O-antigen polysaccharides in strain ST2. **(c)** Validation of glycosylation of carrier proteins FaeG with *S. flexneri* 3a O-antigen polysaccharides in strain ST2. **(d)** Verification of FanC glycoprotein and LPS synthesis in strain ST1 via silver staining and Western blot analysis with anti-His. **(e)** Verification of FaeG glycoprotein and LPS synthesis in strain ST1 via silver staining and Western Blot analysis with anti-His. **(f)** Verification of FanC Glycoprotein and LPS Synthesis Induced by Different Concentrations of Arabinose in ST4. **(g)** Verification of FaeG Glycoprotein and LPS Synthesis Induced by Different Concentrations of Arabinose in ST4. **(h)** Schematic Diagram of the polysaccharide composition of *S. flexneri* 3a, *E. coli* O8 and *E. coli* O157. **(i)** Verification of the glycosylation of O-antigen polysaccharides with *E. coli* O8 and *E. coli* O157 in ST4 using Western blot analysis with anti-His.

To test polysaccharide versatility, two additional O-antigens from *E*. *coli* O8 and O157 were evaluated. Given that the first monosaccharide of *S. flexneri* 3a O-antigen is N-acetylglucoinsamine (GlcNAc), we evaluated the compatibility of two other known O-antigen polysaccharides with different structural features in OMVs delivery vectors. One is the *E. coli* O157 polysaccharide, whose initial monosaccharide is N-acetylgalactosamine (GalNAc); the other is the *E. coli* O8 polysaccharide, which harbors an ABC transport system and consists solely of mannose in its polysaccharide composition (Fig 4H). Plasmids for synthesizing aforementioned O-antigen polysaccharides were introduced into ST3 strain expressing PglL and CTB. Western blot revealed that the polysaccharides were successfully glycosylated to the carrier protein with distinct glycoprotein bands. The ladder-like banding pattern of the glycoprotein synthesized from O-antigen polysaccharide of *E. coli* O157 was similar to that of *S. flexneri* 3a, exhibiting a relatively uniform distribution across lane. In contrast, the ladder-like banding pattern of the glycoprotein derived from O-antigen polysaccharide of *E. coli* O8 is relatively concentrated (Fig 4I). These findings confirm that OMVs-based polysaccharide delivery platform is capable of co-delivering various types of antigens by altering polysaccharide donors and carrier proteins.

### Evaluation of immunological efficacy and safety of OMV-delivered polysaccharides

BALB/c mice were immunized with one of five treatments (PBS, 3a-CTB, OMV-3a-CTB, OMV-3a-LPS, OMV-3a-CTB-LPS) on days 0, 14, and 28. The immunized dose was 30 μg per mouse administered as a single subcutaneous injection for the glycoprotein group, and 1 μg per mouse administered as a single intraperitoneal injection for the OMVs group (Fig 5A). Silver staining and Western blot confirmed target antigen integrity in all formulations (Fig 5B).

**Fig 5:**
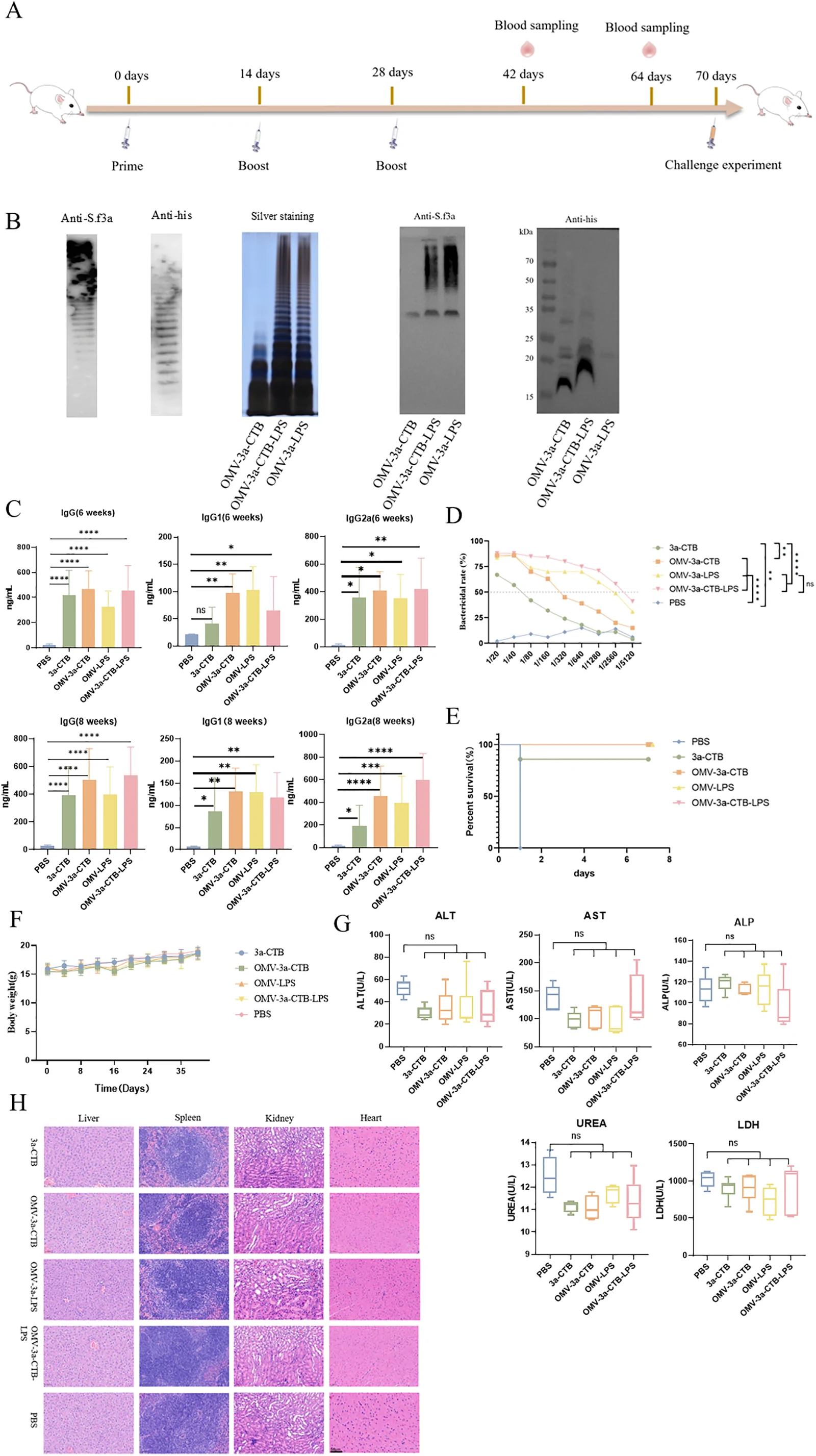
Immunological evaluation of OMV-based vaccine in a murine. **(a)** Schematic timeline of the immunization and challenge study. **(b)** Silver staining and Western blot analysis of glycoproteins and LPS in vaccine 3a-CTB, OMV-3a-CTB, OMV-3a-LPS, and OMV-3a-CTB-LPS. **(c)** ELISA-based measurement of IgG, IgG1 and IgG2a titers against LPS of *S. flexneri* 3a after primary immunizations 42 and 56 days for 3a-CTB, OMV-3a-CTB, OMV-3a-LPS, OMV-3a-CTB-LPS and sterile 1 × PBS groups of BALB/c mice. Differences between groups were evaluated using an unpaired one-way analysis of variance (ANOVA) (****p < 0.0001). **(d)** Serum bactericidal activity was determined on day 42 (*n* = 8). **(e)** Survival of different groups of mice challenged with 5 × 10^7^ CFU per mouse (5 × LD_50_) of *S. flexneri* 3a strain on day 70. Survival was monitored over the subsequent 7 days (*n* = 8). **(f)** Monitored mouse body weight continuously for 42 days following the primary immunization. **(g)** Serum biochemical parameters were monitored 28 days after vaccination, including alanine aminotransferase (ALT), aspartate aminotransferase (AST), alkaline phosphatase (ALP), blood urea nitrogen (BUN), and lactate dehydrogenase (LDH). **(h)** Twenty-eight days after the initial vaccination, the liver, spleen, kidneys, and heart were harvested for histological examination via hematoxylin and eosin (H&E) staining.

At 6 and 8 weeks post-primary immunization, mouse sera were collected via the orbital vein to determine antibody titers against *S. flexneri* 3a lipopolysaccharide by ELISA. Two weeks after the third immunization, all immunized groups exhibited significantly elevated antibody levels relative to PBS control group, with sustained elevation over the subsequent two weeks (Fig 5C).

Next, we evaluated the complement-mediated bactericidal activity of the serum from vaccinated mice. As shown in Fig 5D, serum from mice vaccinated with the OMV-3a-CTB-LPS vaccine displayed robust bactericidal activity against *S. flexneri* 3a strain. Serum from mice vaccinated with OMV-3a-LPS and OMV-3a-CTB also exhibited relatively high levels of bactericidal activity. To validate the protective efficacy of the OMVs delivering the polysaccharide platform in vivo, eight vaccinated mice per group were administered an intraperitoneal injection of approximately 5 × 10^7^ colony-forming units (CFU)/mouse (5 × median lethal dose (LD_50_)) of *S*. *flexneri* 3a at 8 weeks post-primary immunization. After high-dose challenge, the 7-day survival rate was 100% in mice vaccinated with OMV-3a-LPS, OMV-3a-CTB, and OMV-3a-CTB-LPS. The 7-day survival rate of mice vaccinated with 3a-CTB vaccine was 87.5%, whereas mice in the PBS group succumbed within 2 days (Fig 5E and S2 Table). Collectively, these findings demonstrate that OMVs delivering polysaccharides can induce production of corresponding antibodies in vivo, thereby conferring protective efficacy.

After confirming potent immunostimulatory activity of polysaccharide-delivering OMV platform, a preliminary safety evaluation was further conducted. Mice in all vaccine groups underwent standard primary and booster immunization, with continuous monitoring for a designated period to assess vaccine safety. Body weight monitoring revealed no abnormal fluctuations in mice across all vaccine treatment groups throughout the observation period (Fig 5F). Subsequently, serum biochemical parameters were measured 28 days post-vaccination, including alanine aminotransferase (ALT), alkaline phosphatase (ALP), aspartate aminotransferase (AST), blood urea nitrogen (BUN), and lactate dehydrogenase (LDH). All parameters fell within physiological normal ranges, with no significant aberrations detected (Fig 5G). Concurrently, hematoxylin and eosin (H&E) staining of liver, spleen, kidney, and heart tissues revealed no significant histological differences between the vaccine treatment groups and the control group (Fig 5H). These results indicated that the newly established OMV-based polysaccharide-delivering vaccine platform retains potent immunostimulatory activity and exhibits favorable biological safety.

We further validated the long-term protective efficacy of the OMV-delivered *S*. *flexneri* 3a O-antigen polysaccharide vaccine. Antibody titers were measured at 12 weeks post-primary vaccination by ELISA, and results showed that the IgG levels in the mice of the OMV-LPS group were relatively higher, and no statistically significant differences compared to the OMV-3a-CTB and OMV-3a-CTB-LPS groups. In contrast, the IgG2a levels in mice immunized with OMV-3a-CTB and OMV-3a-CTB-LPS groups showed significant differences compared to the OMV-LPS group (S10 Fig).

At 13 weeks post-primary immunization, mice were subjected to intraperitoneal challenge with *S. flexneri* 3a cells at a dose of 5 x 10^7^ colony-forming units (CFU) per mouse (5 × LD_50_). The 7-day survival rate was 100% in mice vaccinated with either OMVs-3a-LPS or OMV-3a-CTB-LPS vaccines. In comparison, the 7-day survival rate was 87.5% for mice vaccinated with OMV-3a-CTB and 62.5% for those vaccinated with 3a-CTB, whereas all mice in the PBS control group succumbed within 2 days (S11 Fig). One-week post-infection, H&E staining was performed on livers and spleens tissues from the mice in challenged group for histological analysis. Compared with the non-inoculated group, no evident histological abnormalities were detected in the vaccinated groups (S12 Fig). Collectively, these findings confirm that the OMV-delivered polysaccharide vaccine platform provides long-term protection and no significant adverse effects in the immunized hosts.

## Discussion

The present study established a novel nanovaccine platform utilizing engineered *S.* Typhimurium OMVs for the co-delivery of bacterial polysaccharides in two distinct forms of LPS and glycoproteins, addressing key limitations in traditional polysaccharide vaccine development. Traditional polysaccharide vaccines suffer from poor immunogenicity (especially in infants and children) and cumbersome chemical conjugation processes, while single-form antigen delivery often fails to elicit comprehensive immune responses. Our platform overcomes these limitations through two key innovations: first, the genetic engineering of the *waaL* gene with an arabinose-inducible promoter, which allows precise temporal control of LPS synthesis without compromising glycoprotein production. This tunable system resolves the intrinsic competition between LPS and glycoprotein biosynthesis observed in wild-type strains, where endogenous O-antigens preferentially conjugate to Lipid A-core [36]. Second, the use of a flagellum- and outer membrane protein-deficient parental strain (Δ*ompACD* Δ*fljB* Δ*fliC* Δ*rfbP*) enhances OMVs purity by eliminating immunogenic contaminants, reducing major potential immunogenic interference from *Salmonella* and ensuring that the immune response is directed specifically at the target polysaccharide antigens. By leveraging OMVs’ inherent adjuvant properties, nanostructural advantages for antigen presentation, and high stability [19,20], this dual-form delivery strategy synergizes the strengths of T-cell-independent (LPS) and T-cell-dependent (glycoprotein) antigens, establishing a flexible foundation for next-generation multivalent vaccines.

The immunological results in BALB/c mice underscored the synergistic efficacy of dual-form polysaccharide delivery, with OMV-3a-CTB-LPS inducing robust humoral immunity, complement-mediated bactericidal activity, and long-term protective efficacy (Fig 5). LPS, as a pathogen-associated molecular pattern (PAMP), activates toll-like receptor 4 (TLR4) signaling in antigen-presenting cells (APCs), priming the innate immune system and enhancing antigen uptake [37]. In contrast, glycoproteins, which are formed by conjugating O-antigens to carrier proteins via PglL-mediated glycosylation, and LPS, which are often covalently linked to outer membrane proteins of bacteria, convert polysaccharides into T-cell-dependent antigens, triggering MHC II-dependent CD4 ⁺ T-cell activation, B-cell class switching, and memory cell formation [38,39]. Our results demonstrated that single-form OMVs formulations (OMV-3a-LPS and OMV-3a-CTB) both conferred 100% survival against a lethal *S*. *flexneri* 3a challenge. OMV-3a-CTB-LPS co-administration increased IgG titer and bactericidal activity, but statistical analysis showed that there was no significant difference. Notably, the sustained IgG and IgG2a production observed at 12 weeks post-immunization confirmed durable immunity (Fig 5) [24]. One limitation of this work is the absence of an irrelevant-antigen OMV negative control. Published studies using the identical Δ*rfbP S*. Typhimurium OMV chassis have shown that empty OMV vector alone cannot elicit detectable heterologous O-antigen-specific IgG despite its intrinsic adjuvant activity [40]. This immunological synergy highlights this platform’s ability to elicit comprehensive and long-lasting protection against bacterial pathogens.

A defining strength of the OMVs platform developed in this work is its versatility, as demonstrated by successful adaptation to diverse carrier proteins and structurally distinct heterologous polysaccharides (Fig 4). Beyond the model carrier protein CTB, we validated the utility of *E. coli* fimbrial proteins FanC and FaeG, both of which underwent efficient glycosylation and co-localization within OMVs. This flexibility allows for the incorporation of pathogen-specific carrier proteins, potentially reducing cross-reactivity and enhancing vaccine specificity, as reported in recent glycoprotein vaccine studies [41]. Additionally, the platform efficiently glycosylated O-antigens from *E. coli* O8 (mannose-only, ABC transport-dependent) and O157 (N-acetylgalactosamine-initiated), confirming compatibility with polysaccharides of varying compositions and biosynthetic pathways. The distinct ladder-like banding patterns observed in Western blot showing uniform for *E. coli* O157 and concentrated for O8 reflected the platform’s ability to accommodate structural differences in polysaccharide chains while maintaining glycosylation efficiency. This inherent versatility enables the platform to facilitate the rapid development of vaccines against a range of multidrug-resistant pathogens, including *Klebsiella pneumoniae*, *Haemophilus influenzae*, and *Streptococcus pneumoniae* [27,41].

Our OMV-based dual-form delivery platform advanced existing nanovaccine technologies to deliver polysaccharide antigens. Previous studies have developed GMMA (Generalized Modules for Membrane Antigens) platforms for polysaccharide delivery [14], but these lack the tunable control of antigen presentation achieved here. Similarly, virus-like particle (VLP)-based conjugate vaccines rely on synthetic assembly, whereas our platform leverages natural bacterial vesicles, reducing production complexity [42]. The arabinose-inducible *waaL* system represented a unique feature, enabling on-demand switching between single-form (glycoprotein-only) and dual-form delivery of LPS and glycoprotein, which could be tailored to specific pathogens or immunization strategies. For example, mucosal pathogens like *Shigella* may benefit from dual-form delivery to elicit both systemic and mucosal immunity, while extracellular pathogens might prioritize LPS-mediated innate activation.

While the present study provides compelling preclinical evidence, several limitations warrant consideration for clinical translation. First, experiments were conducted in a murine model; validation in large animal models (e.g., rabbits, non-human primates) is necessary to evaluate immunogenicity and safety in species more closely resembling humans. Second, the current formulation relies on intraperitoneal administration, and exploring mucosal delivery routes (e.g., intranasal, oral) could enhance local immune responses at pathogen entry sites, which is critical for enteric pathogens like *Shigella* and *Salmonella* [43,44]. Third, while the platform was validated with three *S. flexneri* serotypes and two *E. coli* O-antigens, further testing with a broader range of pathogens (e.g., *N. meningitidis*, *P. aeruginosa*) will confirm its universal applicability. Additionally, the free pentamer CTB can bind to GM1 ganglioside, presenting potential safety risks. The compartmentalization of OMV physically restricts the diffusion of CTB-glycoprotein from the vesicles, which may reduce the off-target binding of gangliosides in the body. However, based on the current data, this risk cannot be completely ruled out. Subsequent experiments may focus on protein antigens that are safer and can elicit immune protection in the body. Despite these limitations, the dual-form OMVs platform represents a flexible, safe, and effective strategy for polysaccharide vaccine development, with the potential to address the global burden of antibiotic-resistant bacterial infections.

## Materials and methods

### Ethics approval and consent to participate

All animal experiments were performed in accordance with institutional and national guidelines for the care and use of laboratory animals, with all efforts made to minimize animal suffering. All animal experiments in this study were reviewed and approved by the Institutional Animal Care and Use Committee (IACUC) of Southwest University. The animal experimental approval number is IACUC-20191120–02. At the experimental endpoint, animals were humanely sacrificed by overdose of anesthetic. A high dose of sodium pentobarbital (200 mg/kg, intraperitoneal injection) was administered. Death was confirmed by permanent cessation of respiration, loss of corneal reflex and absence of pupillary light reflex before tissue collection. General health status including body weight, food and water intake and activity were monitored daily throughout the experiment. Animals showing marked weight loss, lethargy, self-mutilation or obvious pain-related behaviours were intervened or euthanized in advance.

### Bacterial strains, plasmids, and growth condition

All strains and plasmids used in this study were provided in Table 1. Briefly, *E. coli* strain Top10 was used for most molecular biology including plasmid cloning and isolation. *E. coli*, *S. flexneri* and *S.* Typhimurium were grown in Luria Broth (LB) at 37 °C overnight. For plasmid selection, the antibiotics were used at the following concentrations: ampicillin (100 μg/mL), chloramphenicol (12.5 μg/mL), kanamycin (20 μg/mL), and Diaminopimelic acid (50 μg/mL) were added as needed.

**Table 1:** The strains and the plasmids used in this work.

Strains or Plasmids	Description	Source
**Suicide vectors**		
pRE112	sacB mobRP4 R6K ori Cm^+^	Lab collection
**pRE112-D*waaL***	pRE112 vector carrying the *waaL* gene flanked by upstream and downstream gene fragments	This study
**pRE112-D*pagL***	pRE112 vector carrying the *pagL* gene flanked by upstream and downstream gene fragments	This study
**pRE112-*araC*P**_**BAD**_***waaL***	The *waaL* gene inserted into the pRE112-D*pagL* plasmid with an arabinose-regulated promoter	This study
**Recombinant plasmids**		
pACT3	P15a ori, Cm^+^	Lab collection
pACT3–1	pACT3 vector carrying gene *pglL*	This study
pMAL	pUC ori, Amp^+^	Lab collection
pMAL-1	pMAl vector carrying DsbA signal peptide, model protein CTB, and glycosylation site 4573	This study
pMAL-2	pMAl vector carrying ompD signal peptide, model protein CTB, and glycosylation site 4573	This study
pMAL-3	pMAl vector carrying ompD signal peptide, model fimbria protein FanC, and glycosylation site 4573	This study
pMAL-4	pMAl vector carrying ompD signal peptide, model fimbria protein FaeG, and glycosylation site 4573	This study
pYA3337	Asd+ vector, pSC101 ori	Lab collection
pYA3337–1	Asd+ vector carrying genes involved in the biosynthesis of the basic S.f2a O-antigen (galF-gnd), pSC101 ori	Lab collection
pYA3337–2	Asd+ vector carrying genes involved in the biosynthesis of the basic S.f3a O-antigen (galF-gnd), pSC101 ori	Lab collection
pYA3337–3	Asd+ vector carrying genes involved in the biosynthesis of the basic S.f6 O-antigen (galF-gnd), pSC101 ori	Lab collection
pYA3337–4	Asd+ vector carrying genes involved in the biosynthesis of the basic *E. coli* O157 O-antigen (galF-gnd), pSC101 ori	Lab collection
pYA3337–5	Asd+ vector carrying genes involved in the biosynthesis of the basic *E. coli* O8 O-antigen (galF-gnd), pSC101 ori	Lab collection
**E. coli strains**		
χ7232	*E. coli* endA1 hsdR17 (rK ^−^ , mk^+^) glnV44 thi-1 recA1 gyrA relA1 Δ(lacZYA-argF)U169 λpir deoR (ϕ80dlac Δ(lacZ) M15)	Lab collection
χ7213	*E.coli* thi-1 thr-1 leuB6 glnV44 fhuA21 lacY1 recA1 RP4 − 2-Tc::Mu λpir ΔasdA4 Δzhf-2::Tn10	Lab collection
**S. Typhimurium**		
ST1	∆*ompACD* ∆*fljB* ∆*fliC* ∆*rfbP*	Lab collection
ST2	∆*waaL* in ST1	This study
ST3	∆*pagL* in ST2	This study
ST4	∆*pagL*::araCP_BAD_ *waaL* in ST3	This study
**Immunization (S.f3a)**		
3a-CTB	Purified glycoprotein	This study
OMV-3a-CTB	OMV contains glycoproteins	This study
OMV-3a-LPS	OMV contains LPS	This study
OMV-3a-LPS-CTB	OMV contains glycoproteins and LPS	This study
**Strains for challenge**		
0-4584	Wild-type *S. flexneri* 3a strain	Lab collection

### Gene deletion and insertion of *S.* Typhimurium

To construct *S.* Typhimurium strains harbaring optimal glycoprotein synthesis capacity, we employed suicide plasmid-mediated homologous recombination to construct *waaL* gene deletion strain and *waaL* regulae strain [41]. We constructed the required suicide plasmids using the widely used suicide plasmid vector pRE112, which harbors functional elements including SacB, mobRP4, R6K origin of replication (ori), and chloramphenicol resistance (CmR), and *waaL* gene mutations were introduced by positive and negative screening of SacB and resistance.

The recombinant suicide plasmid containing the upstream and downstream fragments of the WaaL gene was introduced into *E. coli* χ7213. A 3:2 mixture of *E. coli* χ7213 containing the plasmid and ST1 strain was plated onto LB solid medium and incubated at 37°C for 24 hours to complete the conjugative transfer. The formed bacterial colonies were evenly spread on LB (CmR) solid culture agar plates and incubated at 37°C for 24 hours until the monoclonal colonies grew. The monoclonal cells were re-inoculated onto LB (CmR) solid culture plates. Well-growing strains were inoculated into LB liquid medium and cultured at 37 °C with shaking until an OD_600_ of approximately 0.3 was reached. The bacterial solution was diluted at ratios of 1:10, 1:100, and 1:1000. A 100μL aliquot of each original and diluted suspension was spread onto 10% sucrose solid medium and incubated at 28°C for 24–36 hours until a single colony emerged. Single colonies that grew well on LB plates but failed to grow on LB with Cm^+^ were verified by PCR.

### Growth curve analysis

To determine whether the growth rate of genetically engineered mutant bacteria had changed, this study used LB liquid medium to plot the growth curves of *Salmonella* and its mutant strains. First, we inoculated each test strain into 5 mL of fresh LB liquid medium and incubated it overnight at 37°C with shaking at 180 rpm. The following day, the overnight bacterial culture was diluted 1:100 and inoculated into 100 mL of fresh LB liquid medium; the culture was then adjusted so that the OD_600_ of the diluted bacterial suspension was approximately 0.05. The bacteria were placed in a 37°C shaking incubator and cultured under continuous agitation at 180 rpm. The OD_600_ value of the bacterial suspension was measured and recorded every hour until the bacteria reached the stationary phase (when the OD_600_ value became relatively stable), and a growth curve was plotted.

### Western blot analyses

*Salmonella* containing target plasmid was grown at 37°C in LB with appropriate antibiotic. When OD_600_ was about 0.6, 0.4 mM IPTG was added and induced at 25°C overnight. After protein expression was induced with IPTG, whole-cell extracts were heated to 100°C with 5 × SDS-PAGE loading buffer (0.6mL 1M Tris-HCl (pH 6.8), 2mL 10% SDS, 0.01g bromophenol blue, 0.5mL β-mercaptoethanol, 2.5mL glycerol and 4.4mL distilled water). Proteins were separated by SDS-PAGE. The glycoprotein was transferred to PVDF membrane, and the mouse source of His-tag labeled monoclonal antibody was used as the primary antibody (Bioworld, 1:8,000 dilution), and the goat anti-mouse IgG labeled by HRP was used as the secondary antibody (Bioworld, 1:20,000 dilution) for Western-blot analysis. Rabbit polyclonal antibody against *S. flexneri* serotype 3a (Tianrun, diluted 1:2,000) was used as the primary antibody, and an HRP-labeled goat anti-rabbit IgG antibody (BioWorld, diluted 1:20,000) was used as the secondary antibody.

### LPS Extraction of *Shigella* strains

The overnight bacterial culture, incubated at 37°C with shaking, was subcultured at a 1:100 dilution and expanded until the optical density at 600 nm (OD_600_) reached approximately 0.9–1.0. Subsequently, the bacterial cells were harvested by centrifugation at 5,500 rpm for 10 minutes. The pellet was resuspended in PBS at a ratio of 10:1 relative to the original culture volume. The resuspended bacterial cells were disrupted by sonication, and the lysate was centrifuged at 12,000 rpm for 30 minutes to obtain the supernatant. To eliminate protein contamination, proteinase K (100 μg/mL) was added and the mixture was incubated at 65°C for 1 hour. Subsequently, RNase (40 μg/mL), DNase (20 μg/mL), 20% MgSO_4_ (1 μL/mL), and CH_3_Cl (4 μL/mL) were added sequentially, followed by overnight incubation at 37°C to eliminate nucleic acid contamination. Phenol preheated to 65°C was added to the sample at a 1:1 (v/v) ratio. The mixture was shaken at 70°C for 30 minutes, then placed in an ice bath for 15 minutes to induce phase separation. After centrifugation at 10,000 rpm for 15 minutes, the upper aqueous layer liquid was carefully collected. Anhydrous ethanol was added to the sample in a ratio of 10:1 (v/v), and the sample was stored at -20 °C for 15 h to precipitate the LPS. The LPS precipitate was collected by centrifugation at 3,000 g for 15 min, then dissolved in ultrapure water. The crude LPS extract after dissolution was loaded into a dialysis bag for dialysis to remove the residual phenol in the aqueous phase. The dialysed LPS was aliquoted and freeze-dried for long-term storage.

### Silver staining of LPS

A 1–2 mL aliquot of the overnight bacterial culture was subjected to centrifugation at 12,000 rpm for 5 min. The bacterial pellet was harvested and resuspended in PBS. The suspension was then centrifuged under the same conditions, and the supernatant was carefully discarded, this cycle was repeat three times. The bacterial pellet was thoroughly resuspended in lysis buffer composed of 1 mL of 0.5 M Tris-HCl (pH 6.8), 0.8 mL of glycerol, 1.6 mL of 10% (w/v) SDS), 0.4 mL of β-mercaptoethanol, and 4.2 mL of distilled water. The suspension was heated to boiling for 10 min and centrifuged for 10 min at 12 000 rpm. 10 μL supernatant was mixed with 90 μL loading buffer composed of 1 mL 0.5 M Tris-HCl (pH6.8), 0.8 mL glycerol, 0.005 g bromophenol Blue, 6.2 mL distilled water, added with 1 μL protease K to digest at 37 °C for 1 h. Subsequently, samples were separated by SDS-PAGE. Following electrophoresis, the gel was fixed overnight in 200 mL of a fixing solution consisting of 25% (v/v) isopropanol and 7% (v/v) acetic acid. Subsequently, the gel was oxidized by immersion in a solution prepared by dissolving 1.4 g periodic acid in 200 mL of distilled water. After through washing with ultrapure water, the gel was stained with a silver staining solution prepared by first mixing buffer A (2.9 mL of 1M sodium hydroxide, 2 mL of ammonia, and 150 mL distilled water), and slowly adding buffer B (1 g of silver nitrate dissolved in 5 mL of distilled water) to buffer A with constant shaking until the solution became clear, and finally the stained gel was developed by immersion in the developer solution containing 0.025 g citric acid and 0.25 g formaldehyde dissolved in 500 mL of distilled water.

### Production and purification of glycosylated proteins

The overnight-cultured bacteria were subcultured at a ratio of 1:100 into to 2 L of fresh medium for further growth. After induction with IPTG, bacteria were harvested by centrifugation at 6,000 rpm for 10 min. The resulting cell pellets were resuspended in 100 mL of binding buffer containing 50 mM Na_2_HPO_4_, 50 mM NaH_2_PO_4_, 10 mM imidazole, and 500 mM NaCl (pH 7.5), followed by cell disruption by sonication. The sonicated lysate was centrifuged at 12,000 rpm for 30 min to remove cell debris. The supernatant was subjected to purification using a Ni-NTA Agarose (QIAGEN) column. Glycoproteins was eluted by elution buffer composed of 50 mM Na_2_HPO_4_, 50 mM NaH_2_PO_4_, 500 mM imidazole, and 500 mM NaCl (pH 7.5). Following verification of the flow-through fractions by Western blot analysis, the correct fractions were pooled and concentrated. The concentrated protein was determined by BCA and the concentration of sugar was determined by anthrone sulfate method.

### OMV isolation and purification

Bacteria induced overnight by IPTG and arabinose were centrifuged at 6000 g for 10 min. The supernatant was collected, and filtered through 0.45 μm filter membrane to further remove bacterial cells. The filtered supernatant was transferred to a 250 mL ultracentrifuge tube, and centrifuged at 40,000 rpm at 4 °C for 3 h. The supernatant was discarded, and the pellet was resuspended in sterile PBS to obtain crude OMVs. The crude OMVs were transferred into a 4 mL ultracentrifuge tube, adjusted to a final volume of 4 mL with PBS, and centrifuged at 40,000 rpm at 4 °C for 3 h. Precipitation was suspended with 800 μL of sterile PBS, and further purified by density gradient centrifugation. Iodixanol was diluted with HEPES buffer to prepare gradients of 10%, 20%, 30%, 40%. The density gradient was layered in a 4 mL ultracentrifuge tube from bottom to top as follows: 40% iodixanol, 30% iodixanol, 20% iodixanol, 10% iodixanol, and finally the OMVs sample. The tube was centrifuged at 4 °C at 200,000 g for 10 h. After centrifugation, the milky band was carefully aspirated and centrifuged again at 4 °C and 200,000 g for 1 h to pellet the OMVs. To remove residual iodixanol, the pellet was resuspended in 1 mL of sterile PBS, yielding purified OMVs.

### Murine immunization and challenge

Every effort was made to minimize animal suffering during the experiments. All mice were acclimated for a period of 7 days following their arrival prior to the commencement of the experiments. All murine immunization procedures were conducted in compliance with all relevant ethical regulations for animal experimentation and were approved by the Institutional Animal Care and Use Committe. Forty 6-week-old BALB/c mice were randomly divided into 5 groups of 8 mice each, and and acclimated to the animal facility for 7 days with free access to sterile water and standard rodent chow. The purified glycoprotein was diluted with PBS and mixed with alum adjuvant. Mice in the first group were subcutaneously immunized with 100 µL solution containing 10 µg of glycoprotein (CTB-3a) on days 0, 14, and 28. Mice in the remaining four groups were intraperitoneally injected with 100 µL solution containing 1 µg OMVs (OMV-CTB-3a, OMV-LPS, OMV-CTB-3a-LPS) on days 0 and 14. Sera were obtained from the mice prior to immunizations and on days 42-, 56-, and 84-day post immunizations. Throughout the immunization process, body weight changes in mice were monitored and recorded regularly. At 8- weeks post-primary immunization, mice were challenged with 5 × 10⁷ CFU/mouse (5 × LD₅₀) of *S*. *flexneri* 3a, and survival was monitored for 7 days.

### ELISA

Blood samples were collected from the posterior orbital venous plexus at 4- and 6-week post-immunization. The samples were allowed to clot at 37 °C for 1 h, and the serum samples were separated by centrifugation at 4,000 rpm for 20 min. LPS antigen of *S. flexneri* 3a and standard antigen were diluted to 2 μg/mL in coating buffer. A 100 μL aliquot of the diluted antigen containing 200 ng were added into each well of 96-well microtilter plates and the plates were incubated overnight at 4 °C. The following day, the wells were washed three times with 200 μL per well of TBST (each liter of solution contains 6.05 g and 9 g NaCl). Non-specific binding sites of the plates were then blocked by adding 200 μL of 3% BCA (bovine serum albumin) at 37 °C for 2 h. The wells were then washed three times with 200 μL of TBST, and the plates were patted dry on absorbent paper. After washing and drying, serum samples were diluted 1:100 in dilution buffer (TBS with 1% BCA), 100 μL of the diluted serum was added to each well and incubated at 37°C for 1 h. After another washing and drying step, biotin-labeled goat anti-mouse IgG, IgG1 and IgG2a secondary antibodies were diluted 1:5,000 in TBST, and 100 μL each well was added to the respective wells, and incubated at 37 °C for 50 min. After another three washes with TBST and drying, 100 μL of the streptavidin-alkaline phosphatase conjugate, diluted 1:3,000 in TBST, was added to each well, and the plate was incubated at 37 °C for 40 min. Finally, 100 μl aliquot of color solution (PNPP, 4-Nitrophenyl phosphate disodium sail hexahydrate) was added to each well, and the plate was incubated in the dark at 37 °C for 15 minutes to allow color development. The reaction was terminated by adding 50 μL of 3 M NaOH stop solution to each well, and the absorbance was measured at a wavelength of 450 nm using a microplate reader.

### Serum bactericidal assay

*S. flexneri* 3a was cultured overnight at 37°C with shaking. The overnight culture was subcultured at a 1:100 dilution into fresh LB broth and incubated until the optical density at 600 nm reached approximately 0.8. The bacterial suspension was then diluted to a concentration of 2.0 × 10^4^ CFU/mL. The sera from each group of mice were pooled and heated at 56 °C for 30 min in a water bath to inactivate the complement components. The inactivated sera were serially diluted to the required concentrations and 20 μL aliquots of each dilution were added to each well. Subsequently, 10 μL of the bacterial suspension was added to each well, and the mixture was incubated at room temperature for 1 h.

Next, 20 μL of fresh rabbit complement was added to each well, and the plate was incubated at 37°C for 1 h to allow bactericidal activity. After incubation, 100 μL aliquots of the mixture were serially diluted 10-fold in sterile PBS, and 100 μL of each dilution was spread onto LB agar plates, The plates were cultured overnight at 37°C to allow colony formation. The serum bactericidal activity was calculated by counting the number of bacteria on the plate.

## Supporting information

S1 Table Quantitative detection of LPS. We expanded each group of bacteria to a culture volume of 100 mL in liquid medium, and then added arabinose to induce the expression of WaaL in the latter two groups. Each group of LPS was dissolved to a final concentration of 2 mg/mL to prepare the test solution.
(TIFF)

S2 Table Survival of different groups of mice challenged with 5 × 107 CFU per mouse (5 × LD_50_) of S. flexneri 3a strain on day 70. Survival was monitored over the subsequent 7 days (n = 8).
(TIFF)

S1 Fig PCR identification of the ST1-missing glycosyltransferase *waaL* gene. Lane 11: strain ST1 negative control. Lane 12: plasmid pRE112-DwaaL positive control.
(TIFF)

S2 Fig Verification of the inability of strain ST2 (deficient in *waaL* and *rfbP*) to synthesize O antigen polysaccharide of *S. flexneri* 3a. (TIFF)

S3 Fig Verification of the pentameric structure of CTB by Western blot technique. Lane 1: Treatment of CTB samples with non-reducible buffer solution. Lane 2: Treatment of CTB samples with reducible buffer solution.
(TIFF)

S4 Fig Detection of *S. flexneri* 2a, 6 glycoprotein expression in strain ST2. Among them, the signal peptide of the carrier protein is DsbA.
(TIFF)

S5 Fig Detection of *S. flexneri* 2a and 6 glycoprotein expression in strain ST2. Among them, the signal peptide of the carrier protein is OmpD.
(TIFF)

S6 Fig Identification of *S. flexneri* 3a Glycoprotein in OMV-3a-CTB. (TIFF)

S7 Fig A. PCR verification of pagL gene deletion. B. PCR verification of the insertion of the *waaL* gene containing an arabinose-inducible promoter.
(TIFF)

S8 Fig Verification of glycoprotein and LPS synthesis in strain ST4(pYA3337-2) with and without arabinose using silver staining and Western Blot analysis with anti-His and anti-*S. flexneri* serotype 2a sera. (TIFF)

S9 Fig A. Verification of the glycosylation pattern of the carrier protein FanC and *S. flexneri* 3a O-antigen polysaccharide in the outer membrane protein of the ST2 strain. B. Verification of the glycosylation pattern of the carrier protein FaeG and *S. flexneri* 3a O-antigen polysaccharide in the outer membrane protein of the ST2 strain.
(TIFF)

S10 Fig ELISA-based measurement of IgG, IgG1 and IgG2a titers against *S. flexneri* 3a strain LPS after primary immunizations 84 days for 3a-CTB, OMV-3a-CTB, OMV-3a-LPS, OMV-3a-CTB-LPS and sterile 1 × PBS groups of BALB/c mice. Differences between groups were evaluated using an unpaired one-way analysis of variance (ANOVA) (****p < 0.0001).
(TIFF)

S11 Fig Survival of different groups of mice challenged with 5 × 10^7^ CFU per mouse (5 × LD_50_) of *S. flexneri* 3a strain on day 70. Survival was monitored over the subsequent 7 days (n = 8).
(TIFF)

S12 Fig H&E staining confirmed that no significant pathological changes occurred in the tissues of immunized mice following infection with *S. flexneri* 3a. (TIFF)

S1 File Zip archive containing unprocessed raw original gel and immunoblot images underlying key figures of this study. Contains raw source images for silver-staining gels, Coomassie Brilliant Blue staining gels and Western blot, corresponding to main text figures and supplementary figures.
(PDF)

S2 File Fig Abstract. (TIFF)
