# Supplementary material for: Modular design of Salmonella Typhimurium OMV-based nanovaccines for tunable dual-form polysaccharide antigen delivery
Source: PLoS Pathog. 2026 Nov 4;22(11):e1014781. doi: 10.1371/journal.ppat.1014781 (PMC13759075; doi:10.1371/journal.ppat.1014781)

Figure 1d (left)   Figure 1f (right)

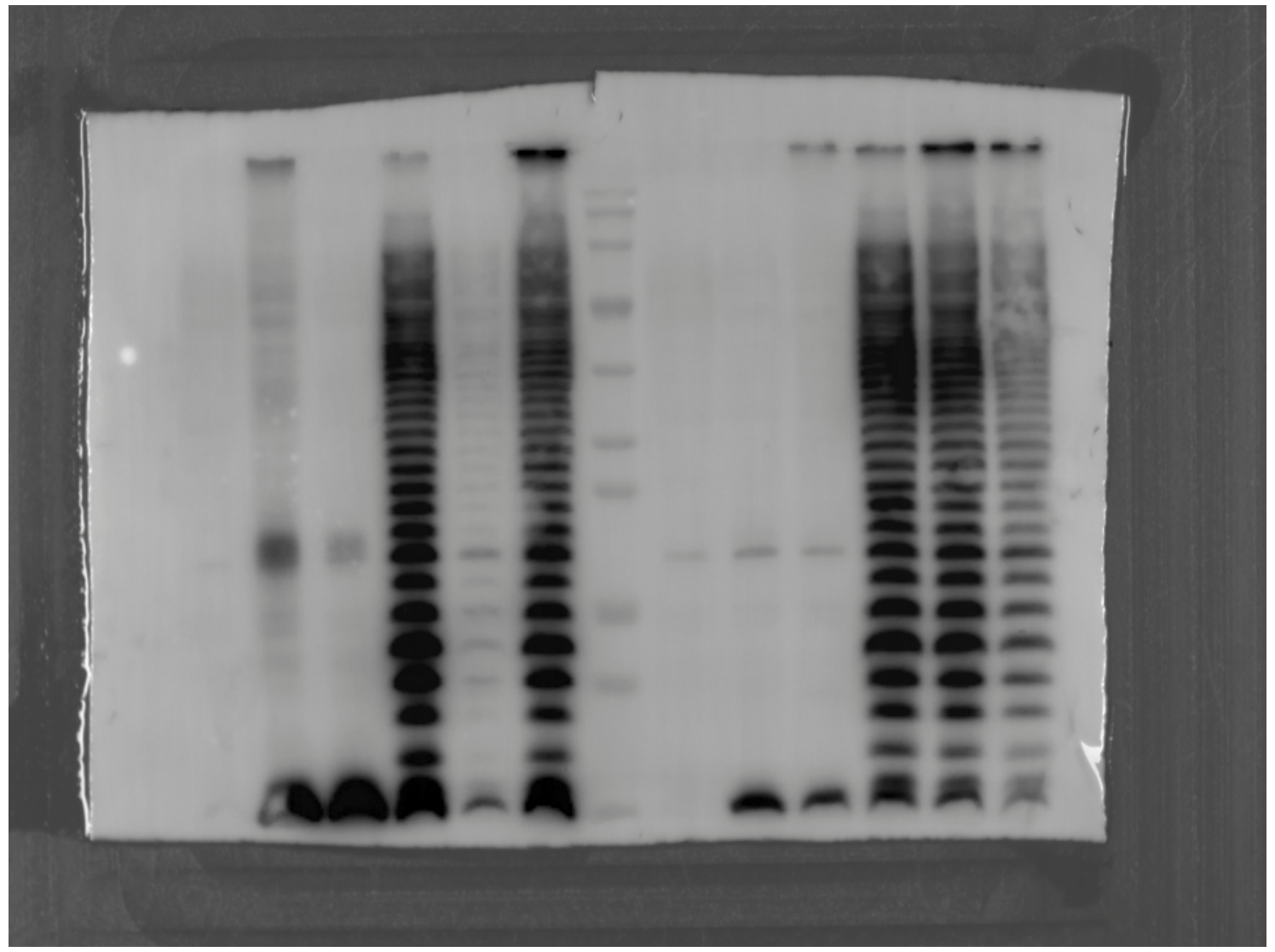

Figure 1f

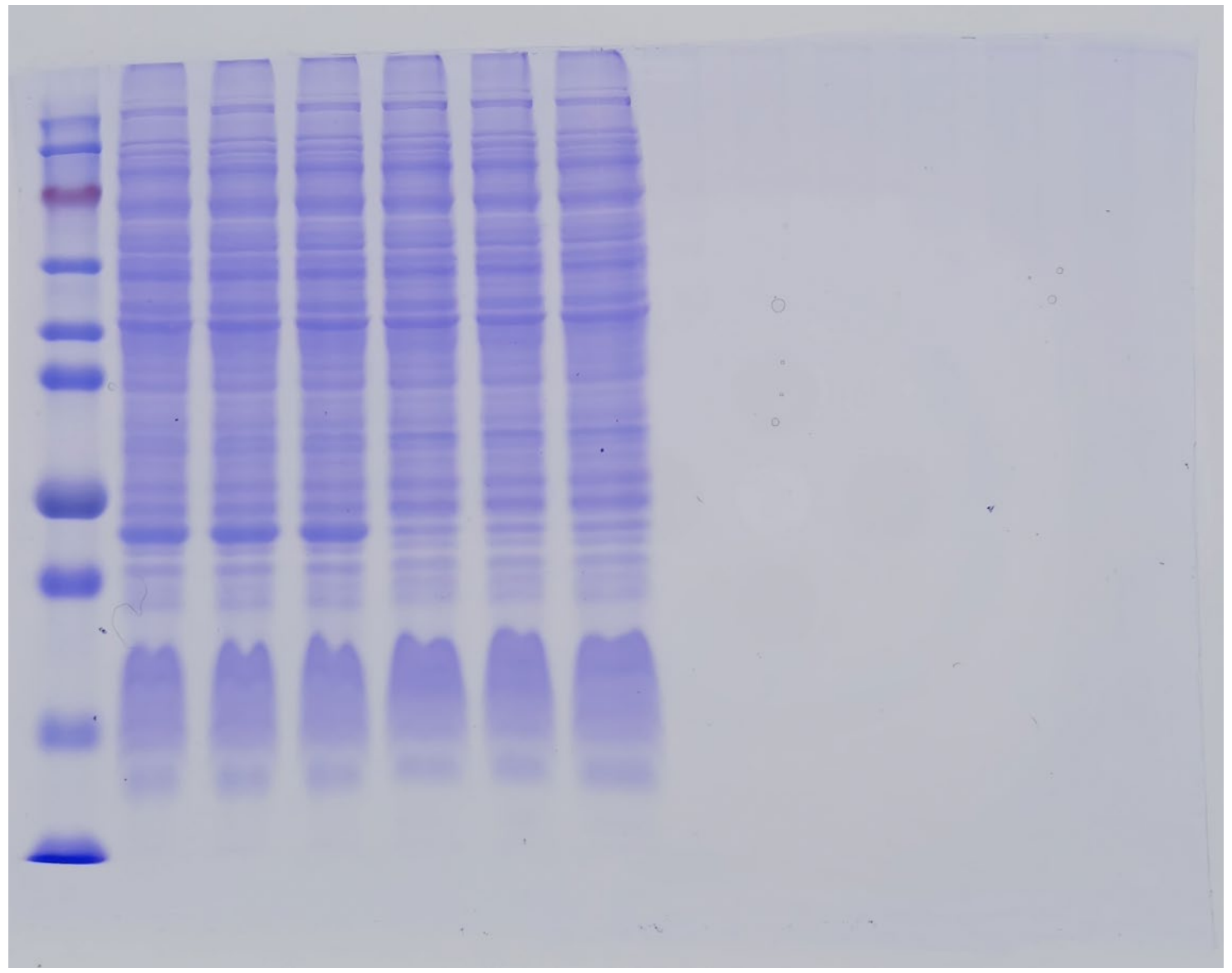

Figure 2b (left) Figure 2a (right)

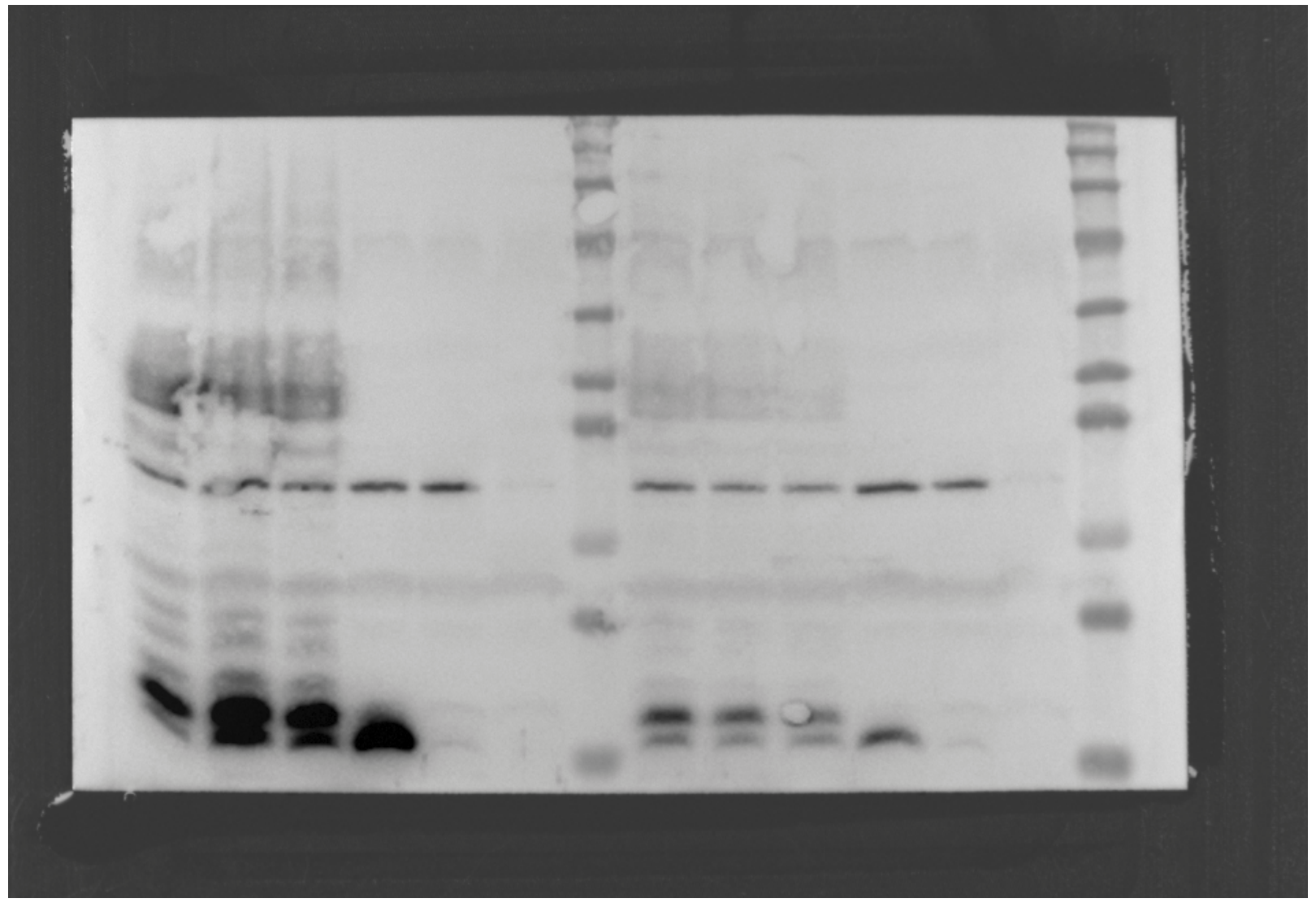

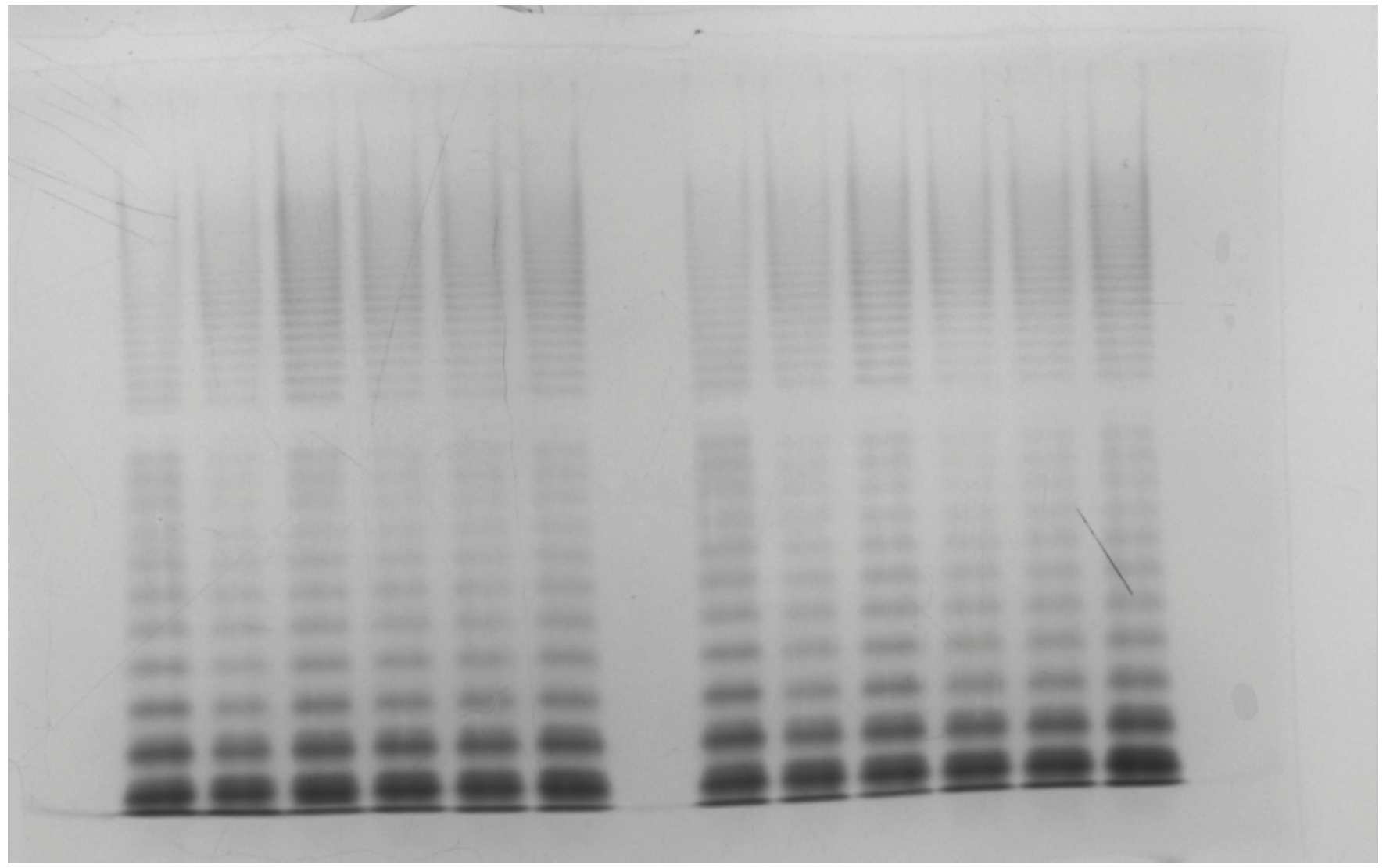

Figure 2c (left) Figure 2d (right)

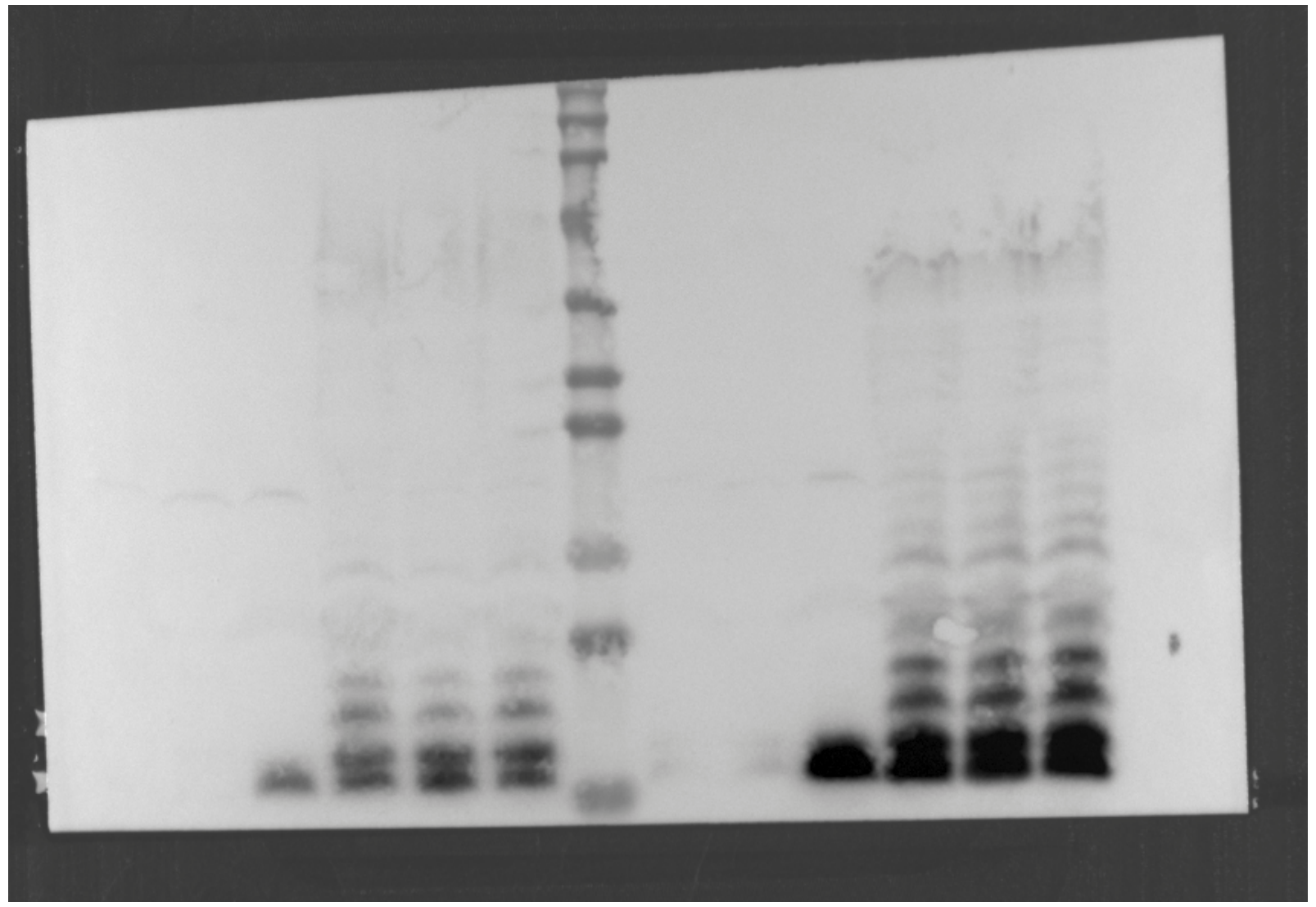

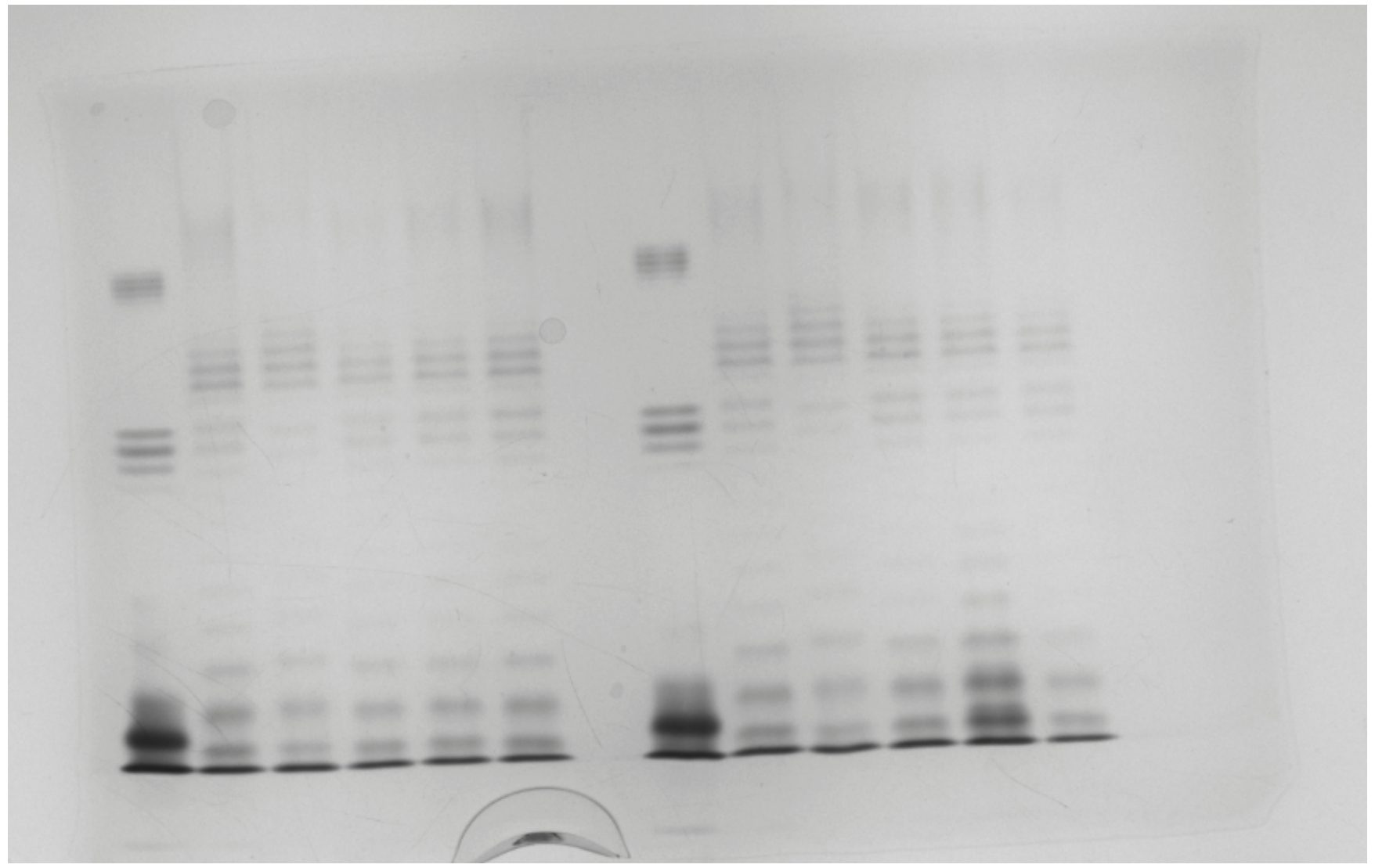

Figure 2e

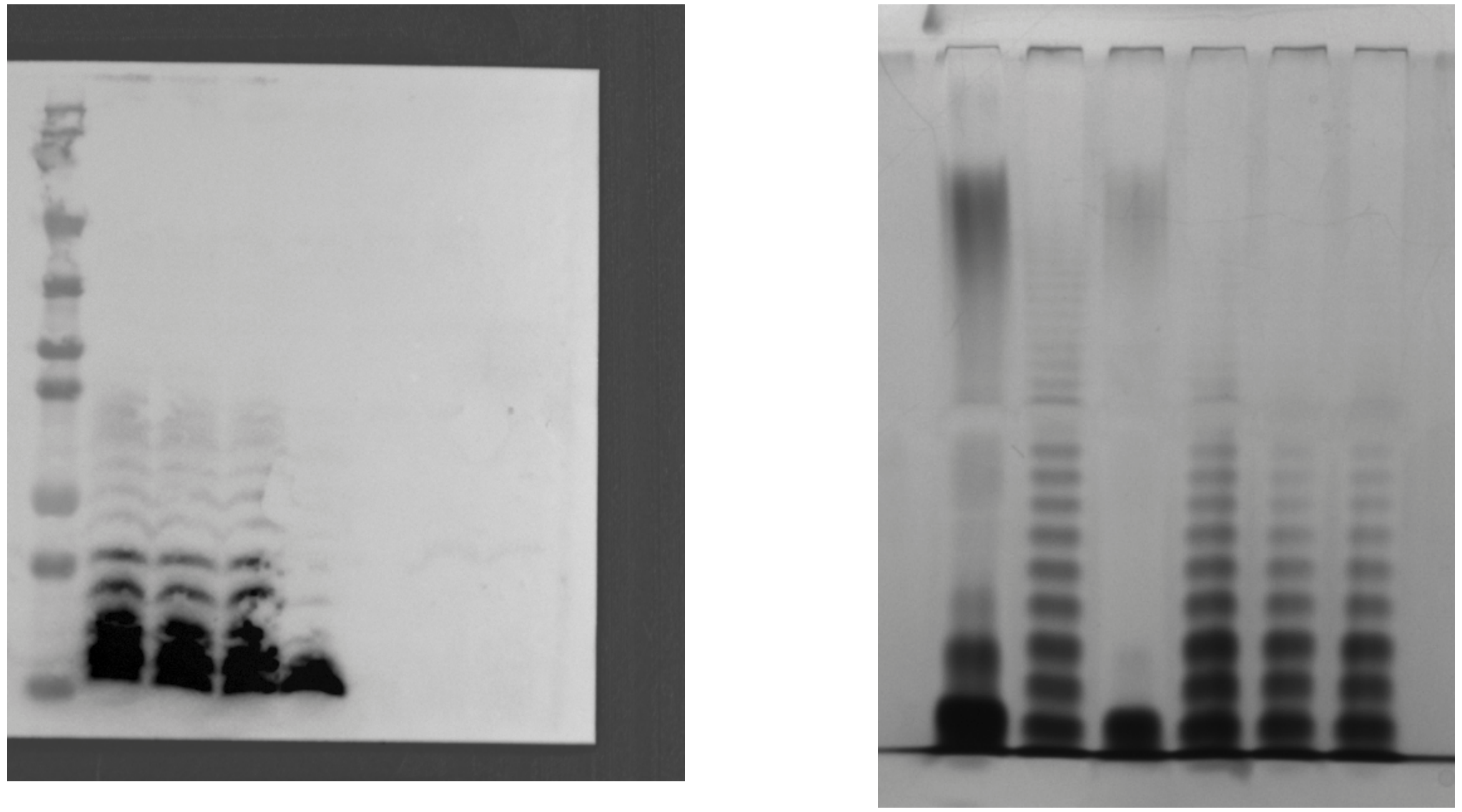

Figure S8: Lane 1-7   Figure 3c: Lane 8-10

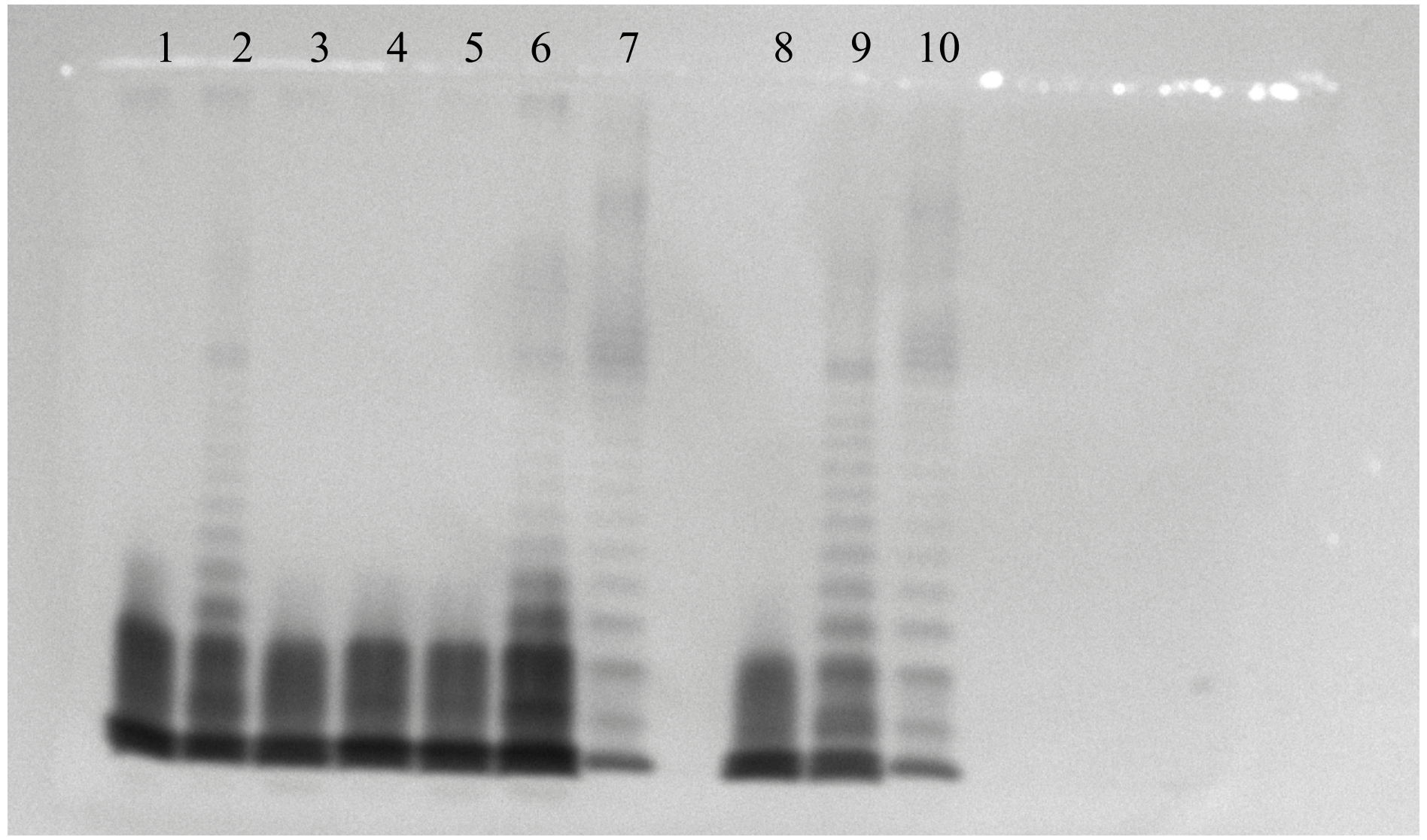

Figure 3c: Lane 1-3

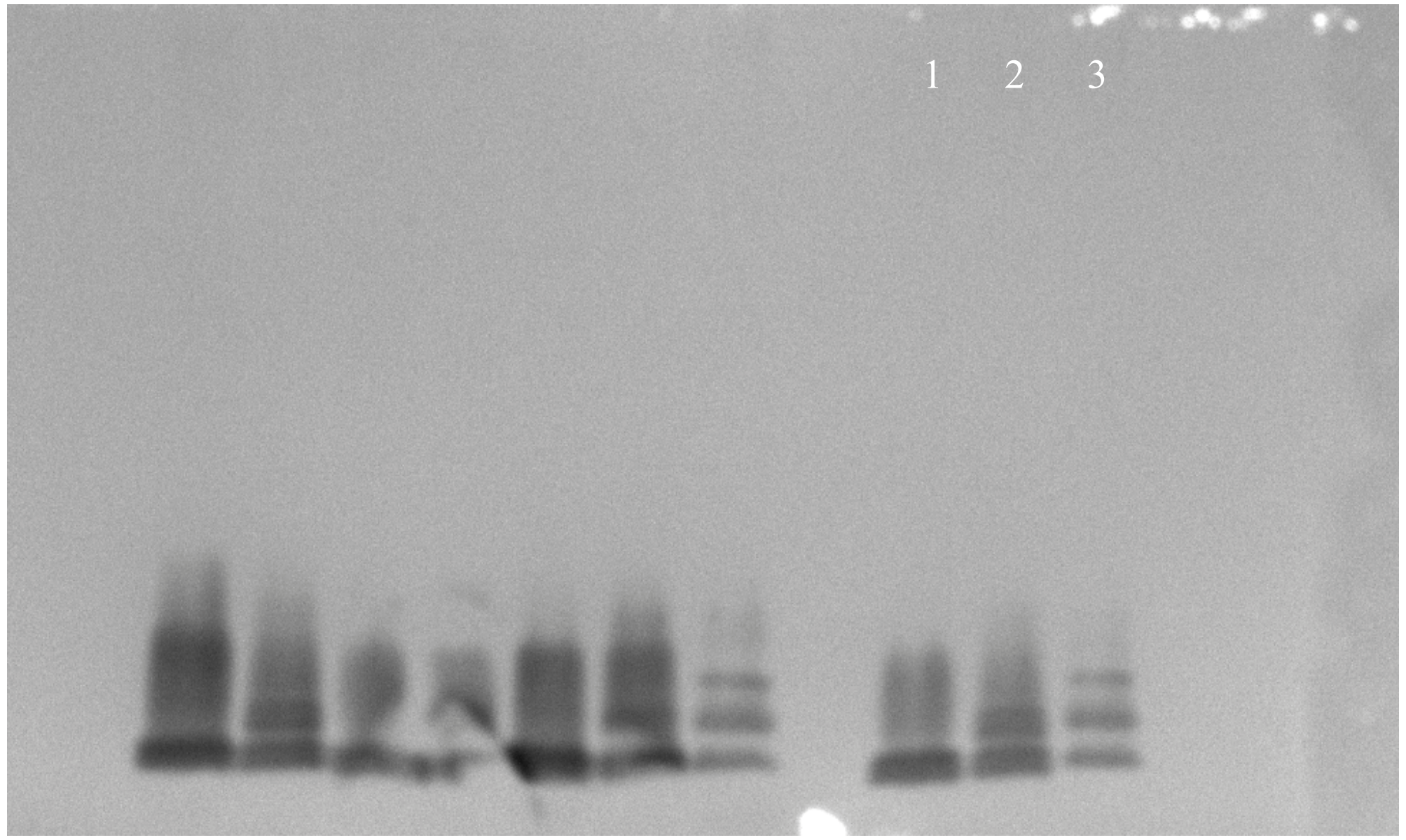

Figure 3c: Lane 1-3

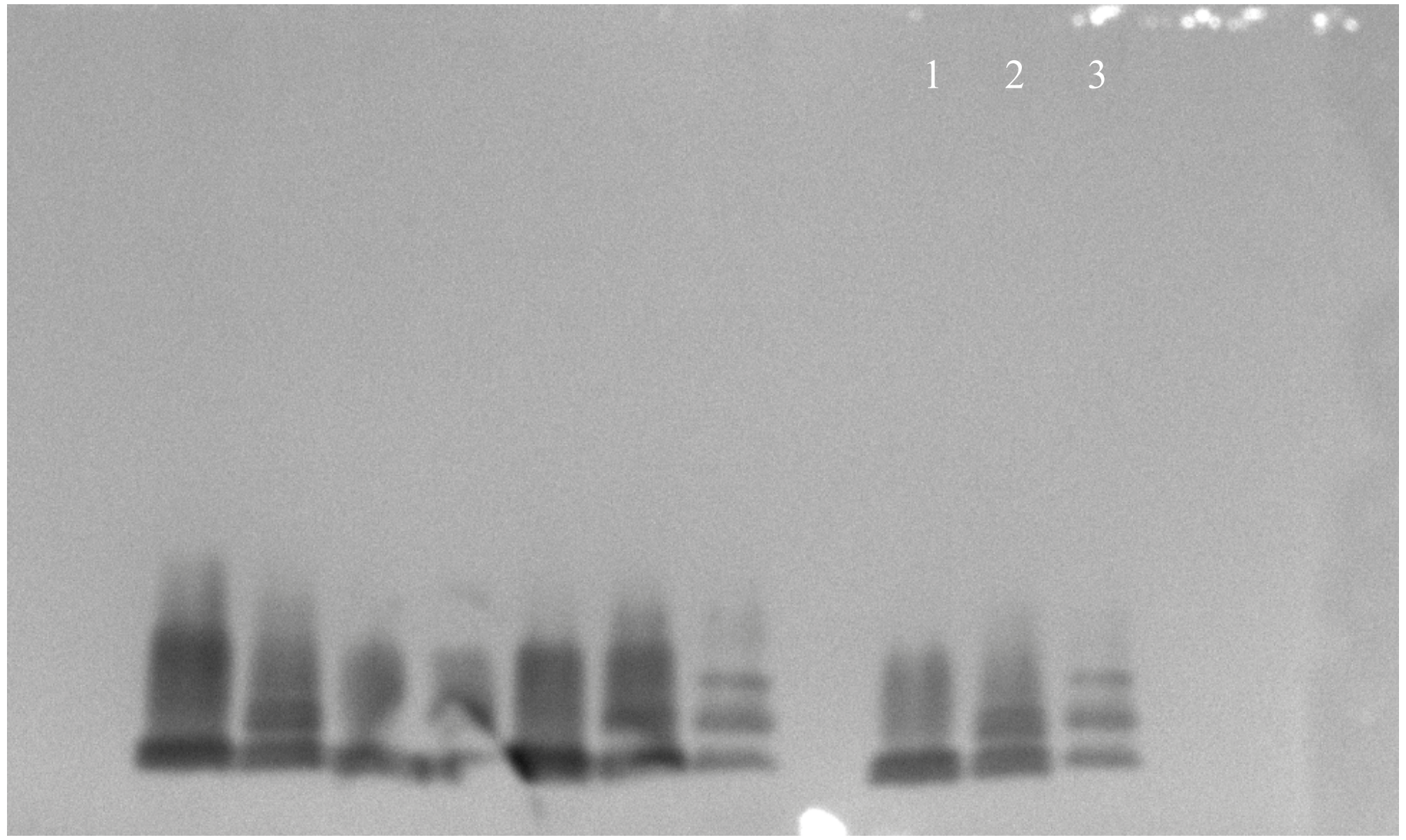

Figure 3f: Lane 1-8   Figure 3c: Lane 9-11

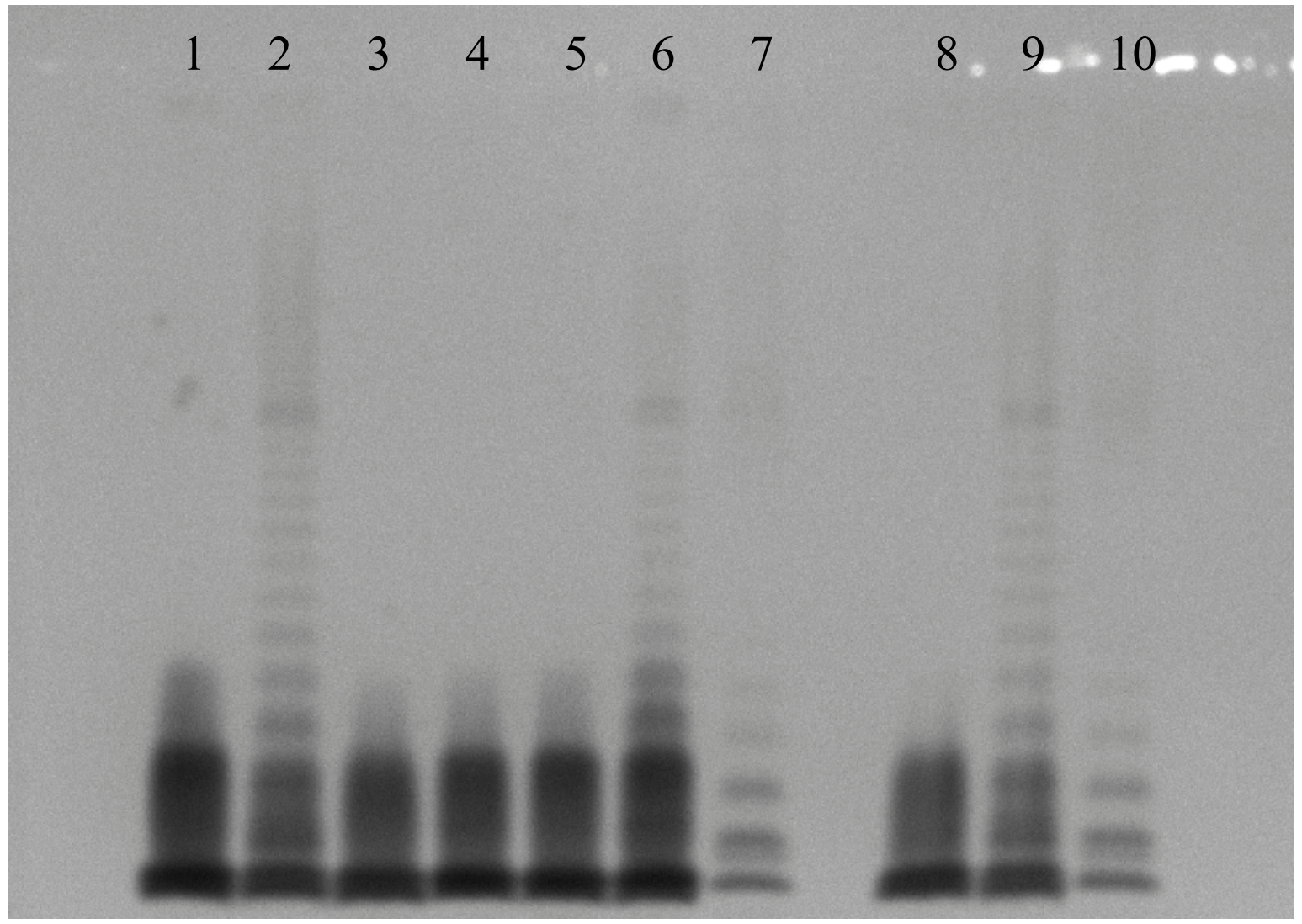

Figure 3c: Lane 1-3

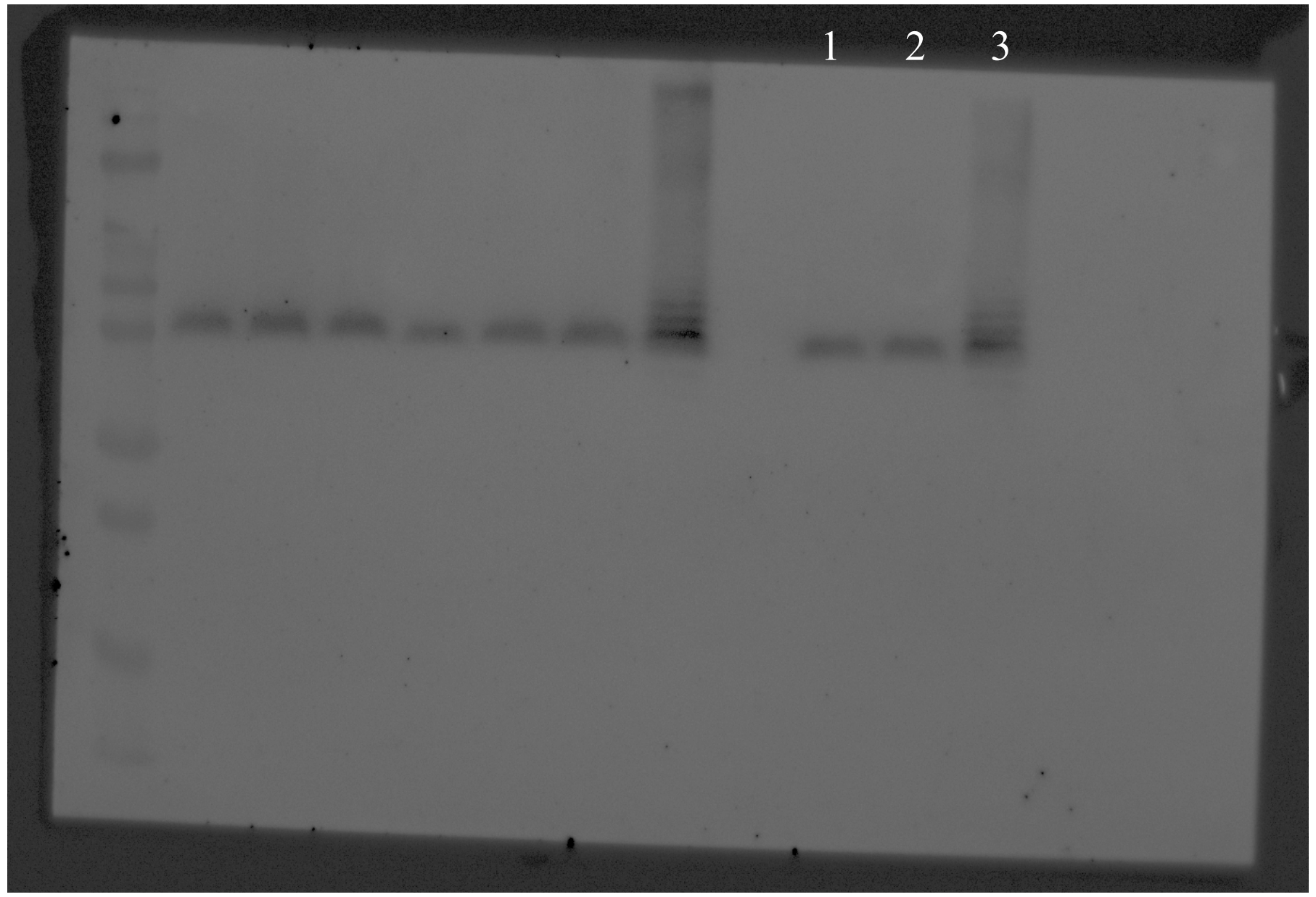

Figure 3c: Lane 1-3 Figure 3f: Lane 4-10

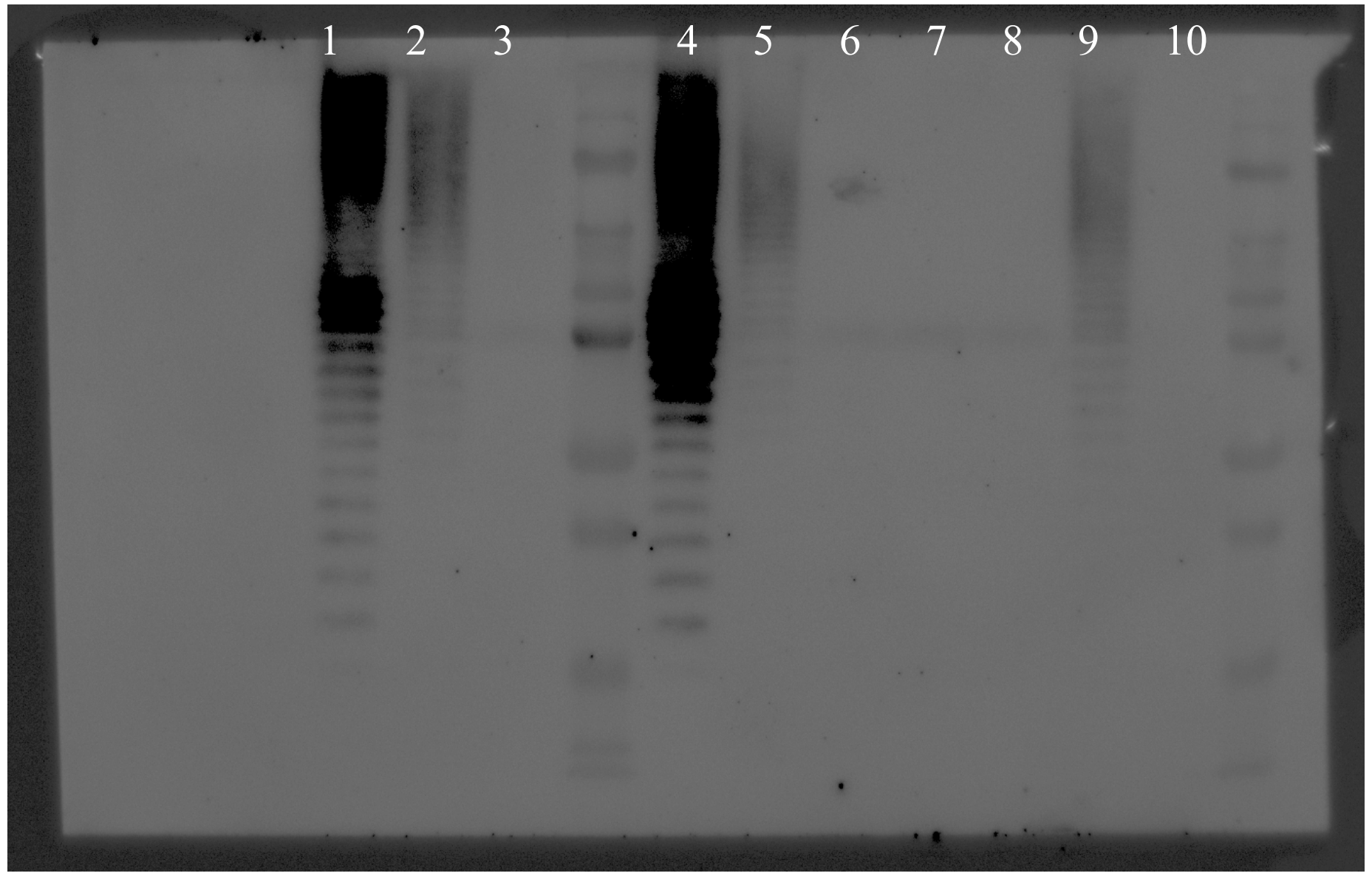

Figure 3c: Lane 1-3 Figure 3f: Lane 4-10

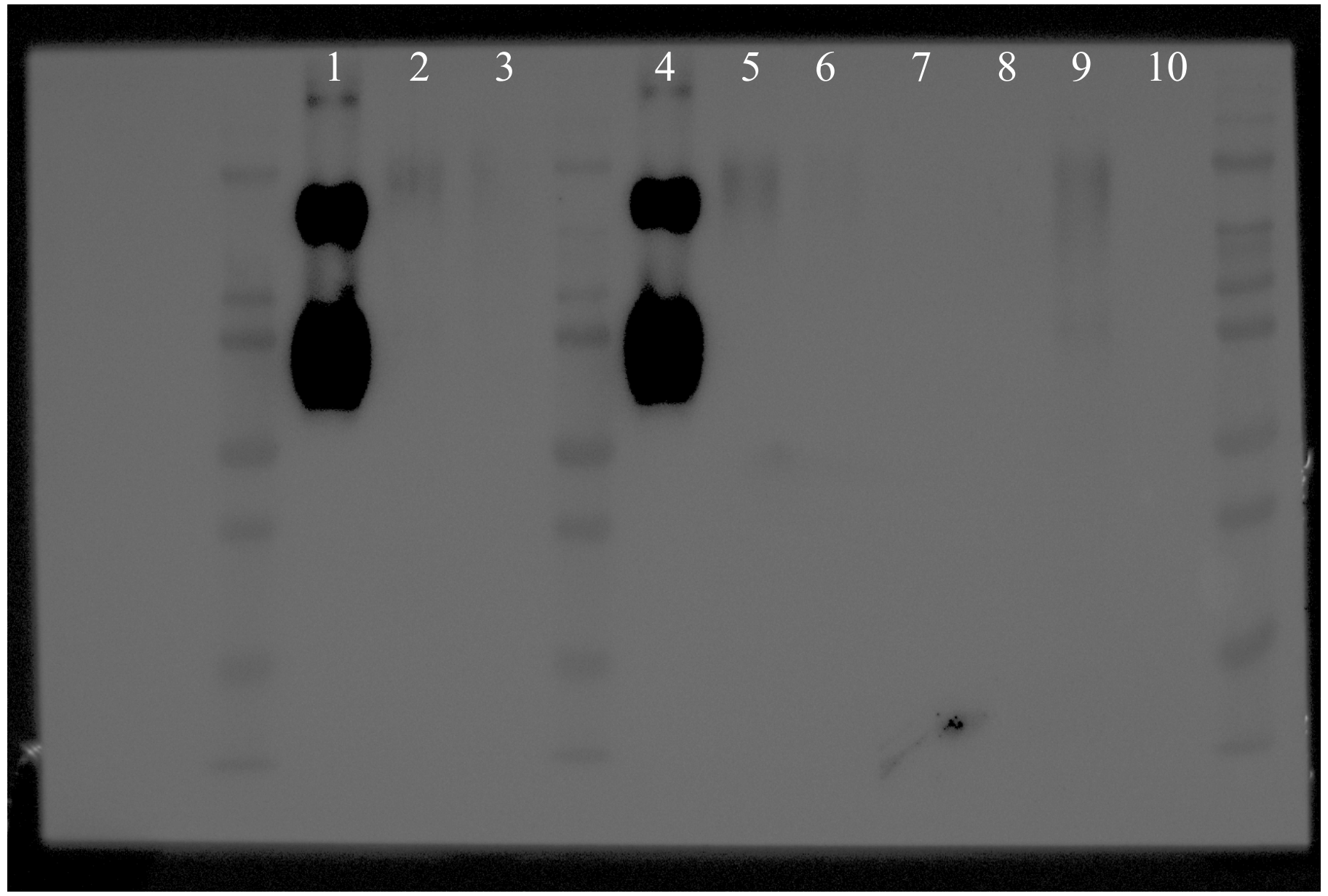

Figure 3f (right)   Figure S8 (left)

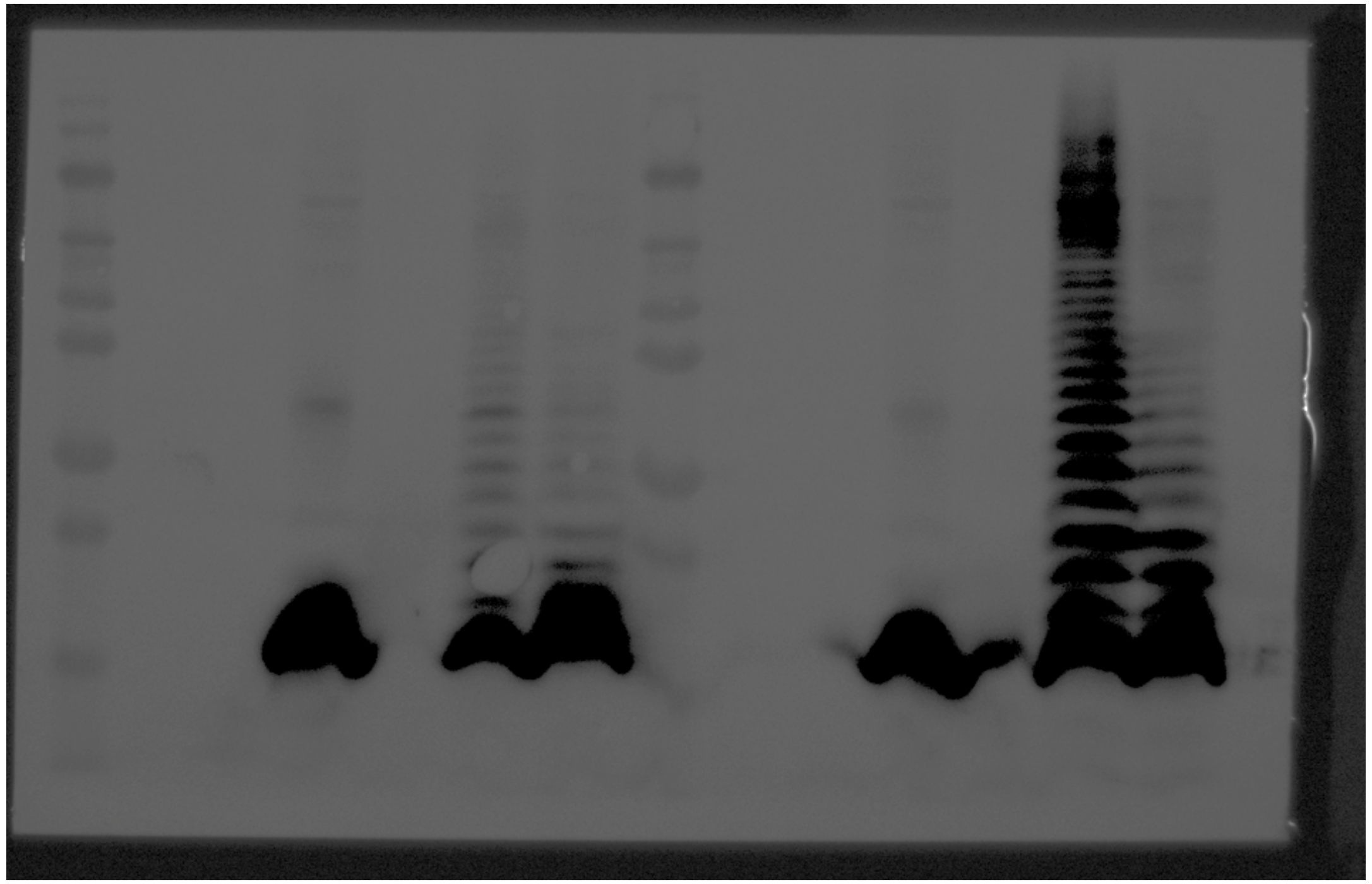

Figure 3f (right) Figure S8 (left)

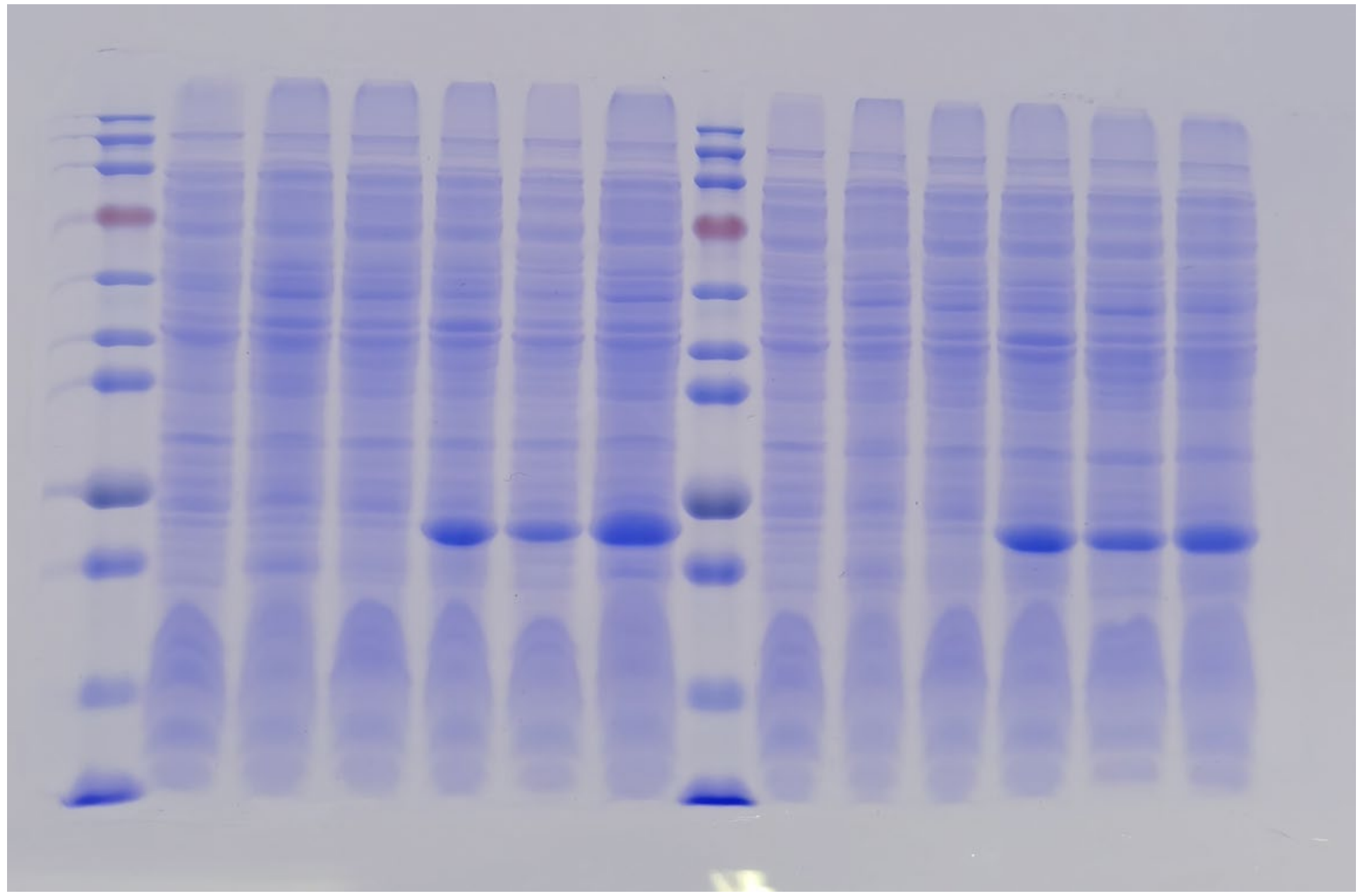

Figure 4a: Lane 1-3

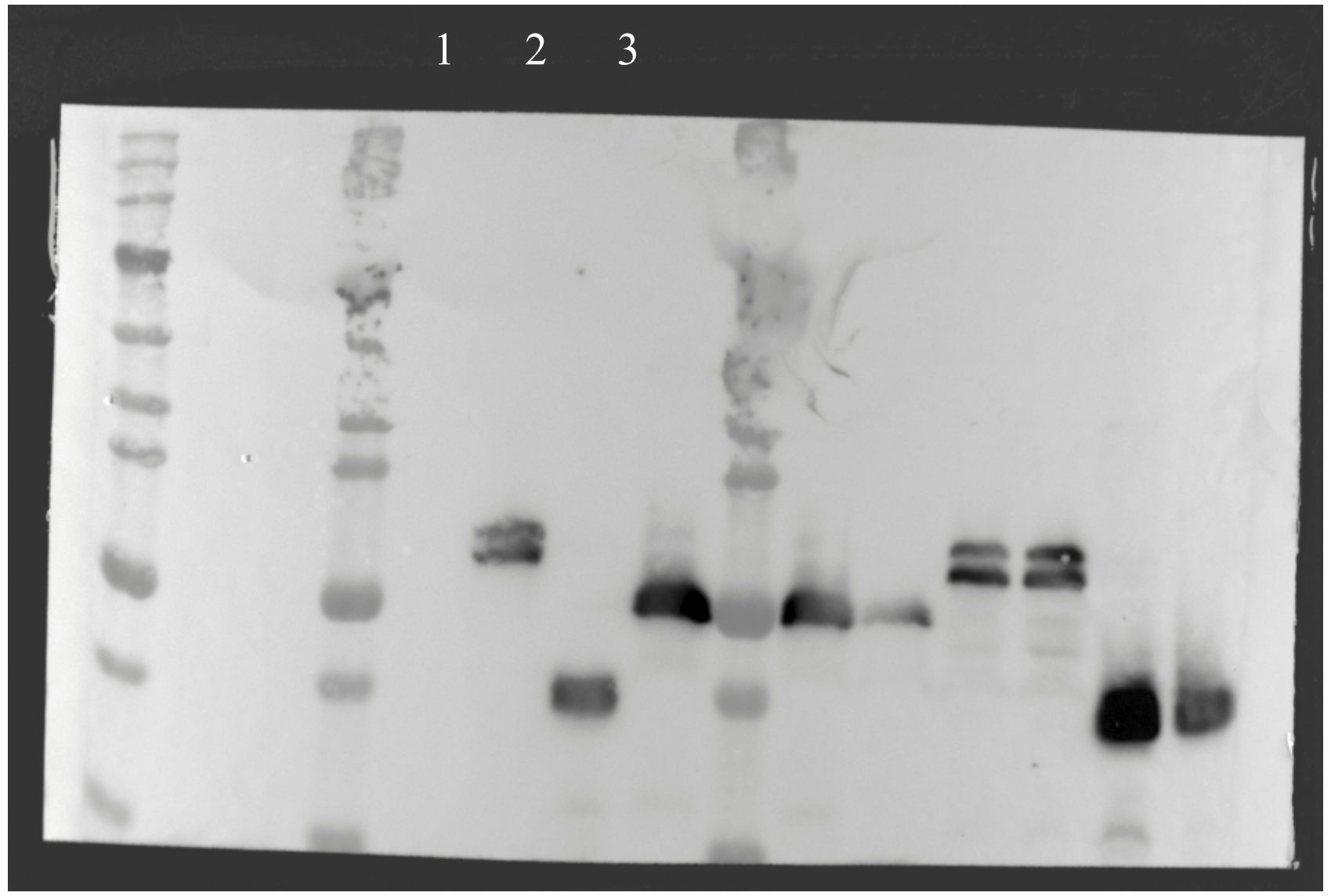

Figure 4b (right)

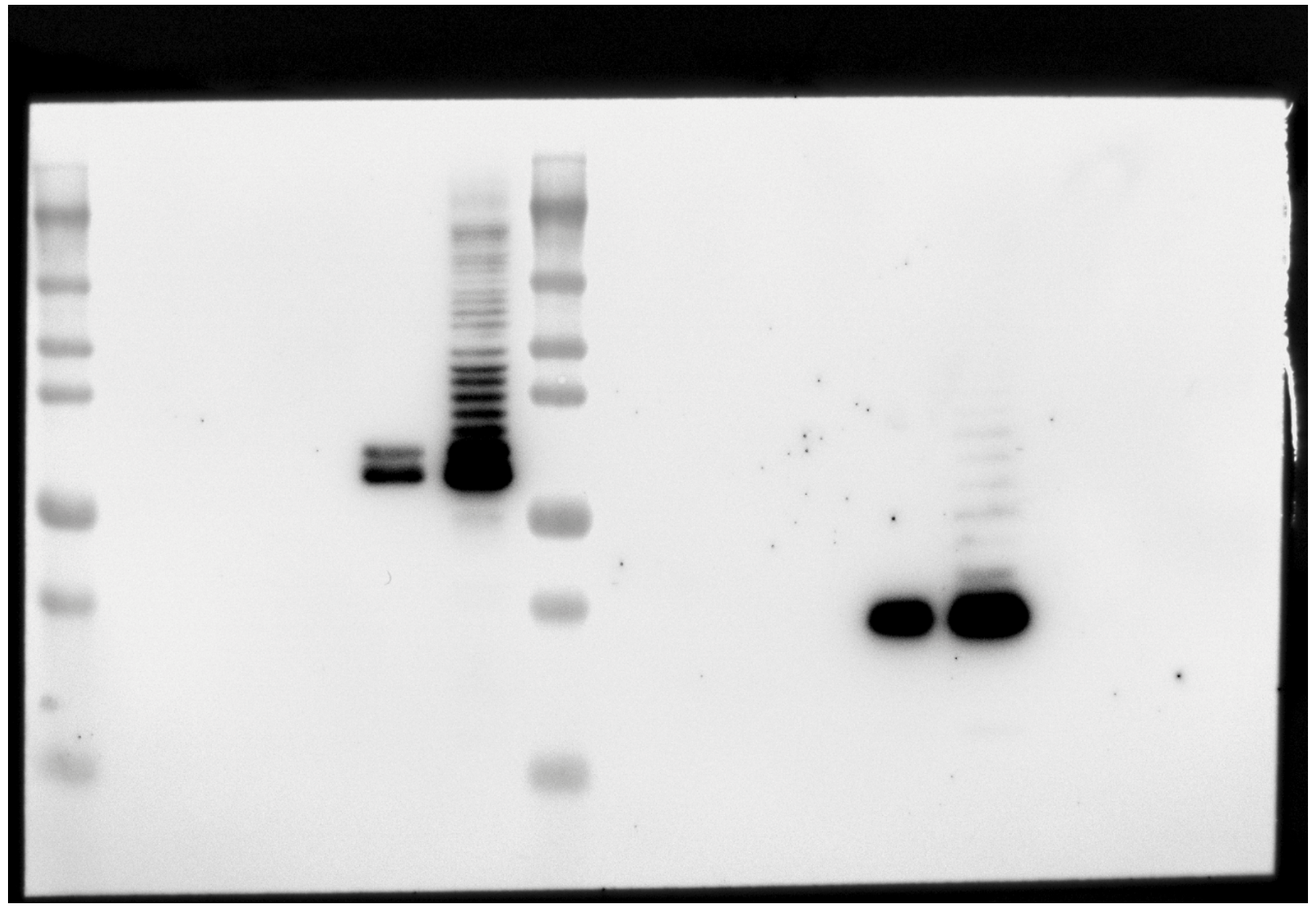

Figure 4c (left)

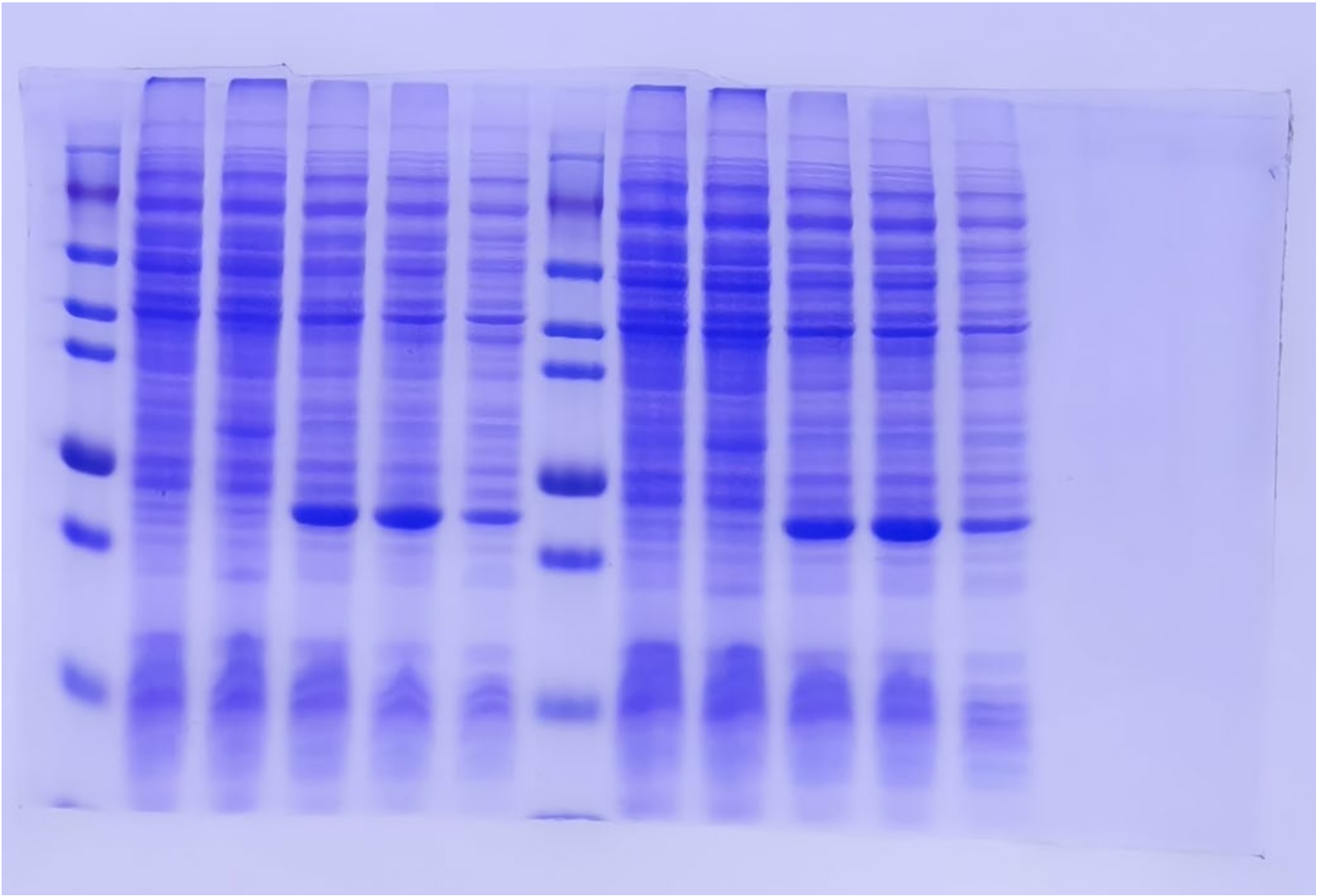

Figure 4e (left) Figure 4d (right)

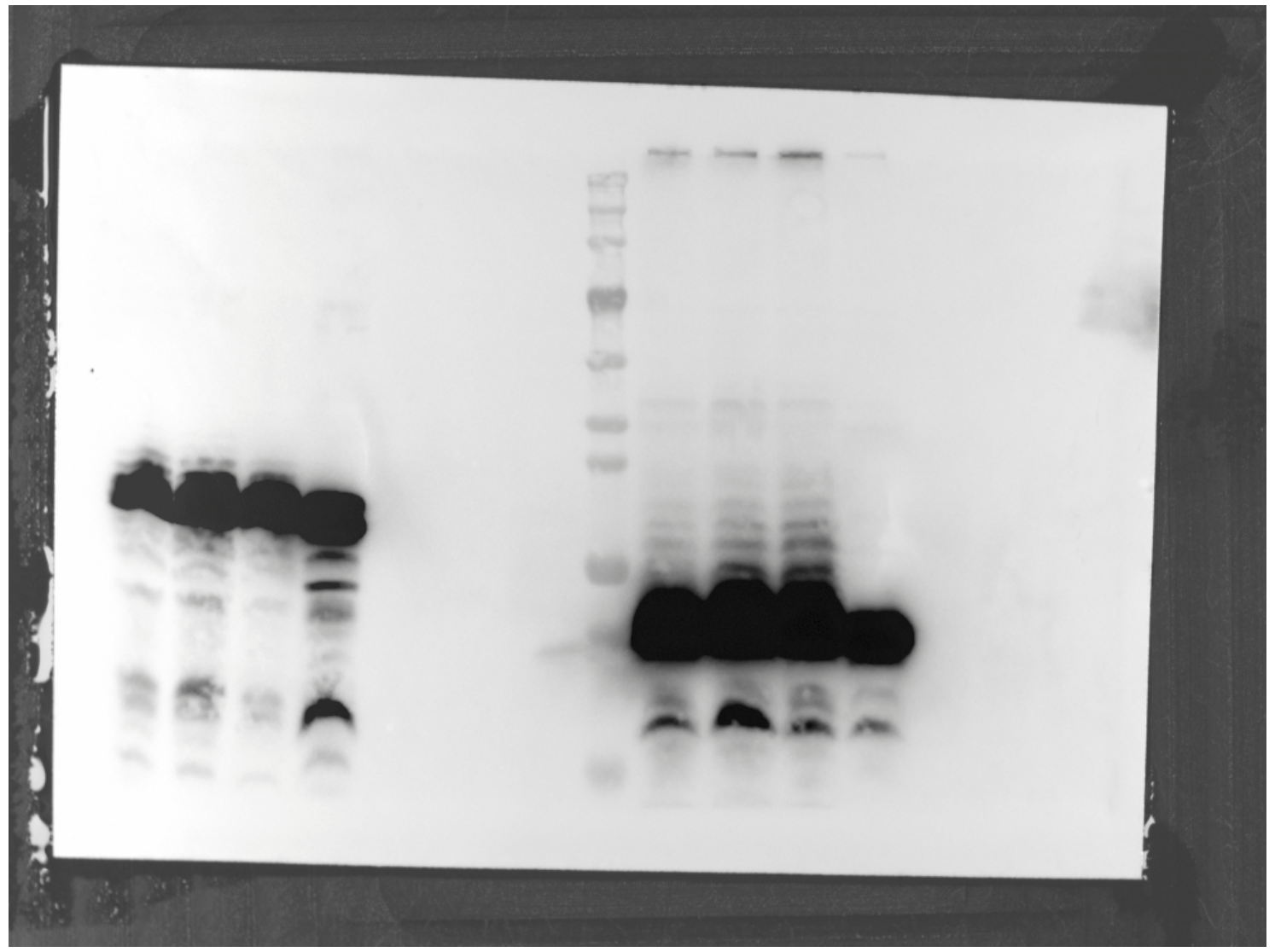

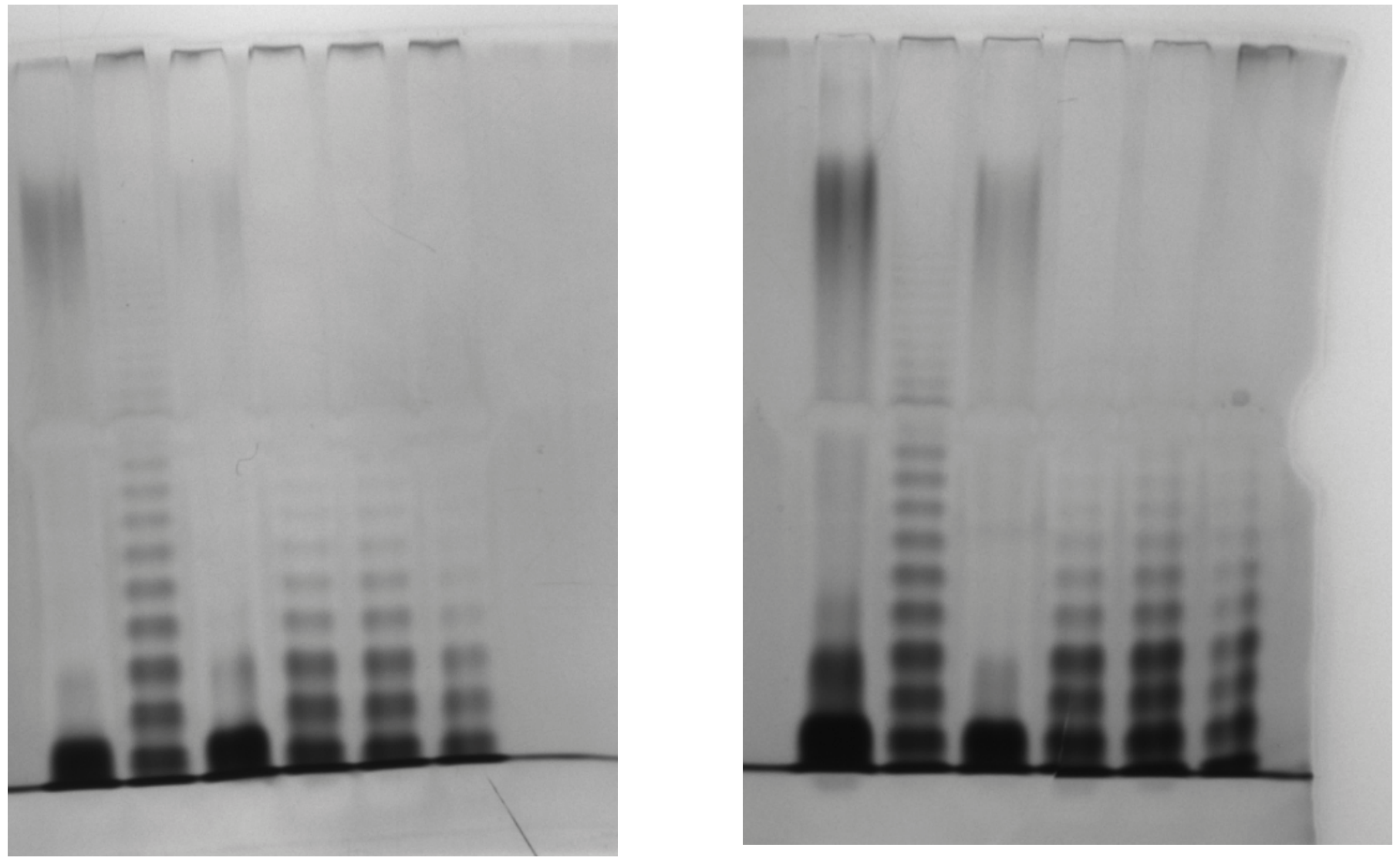

Figure 4f

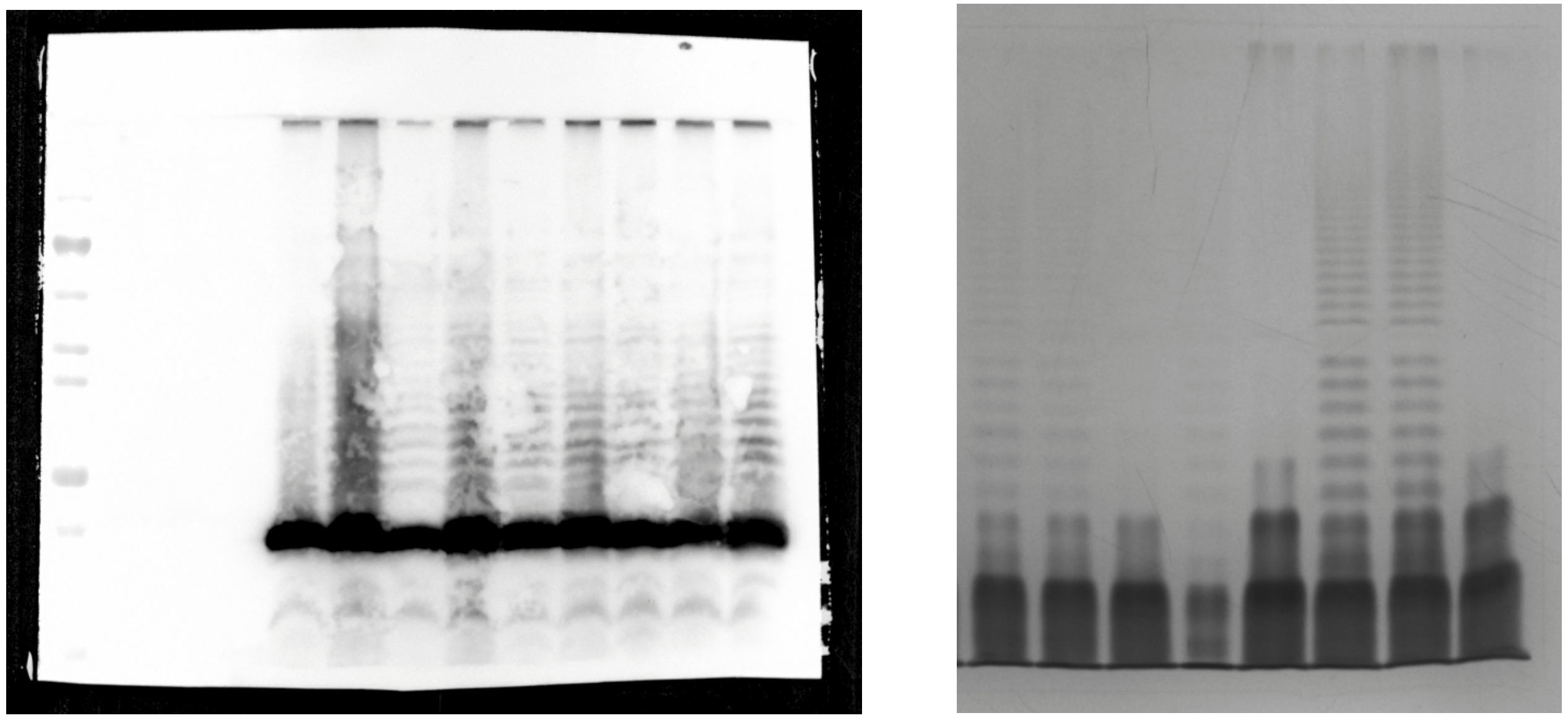

Figure 4g

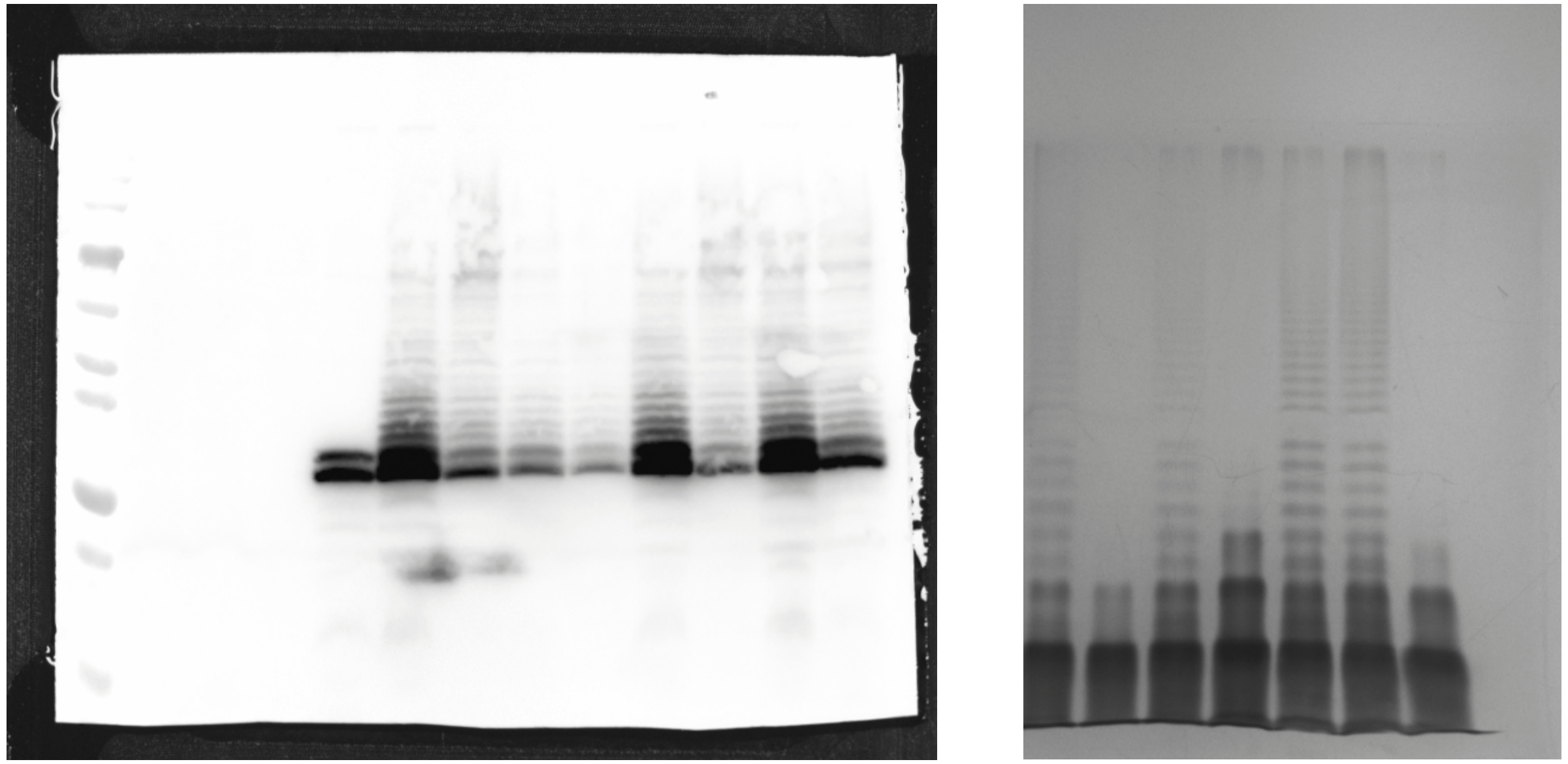

Figure 4i

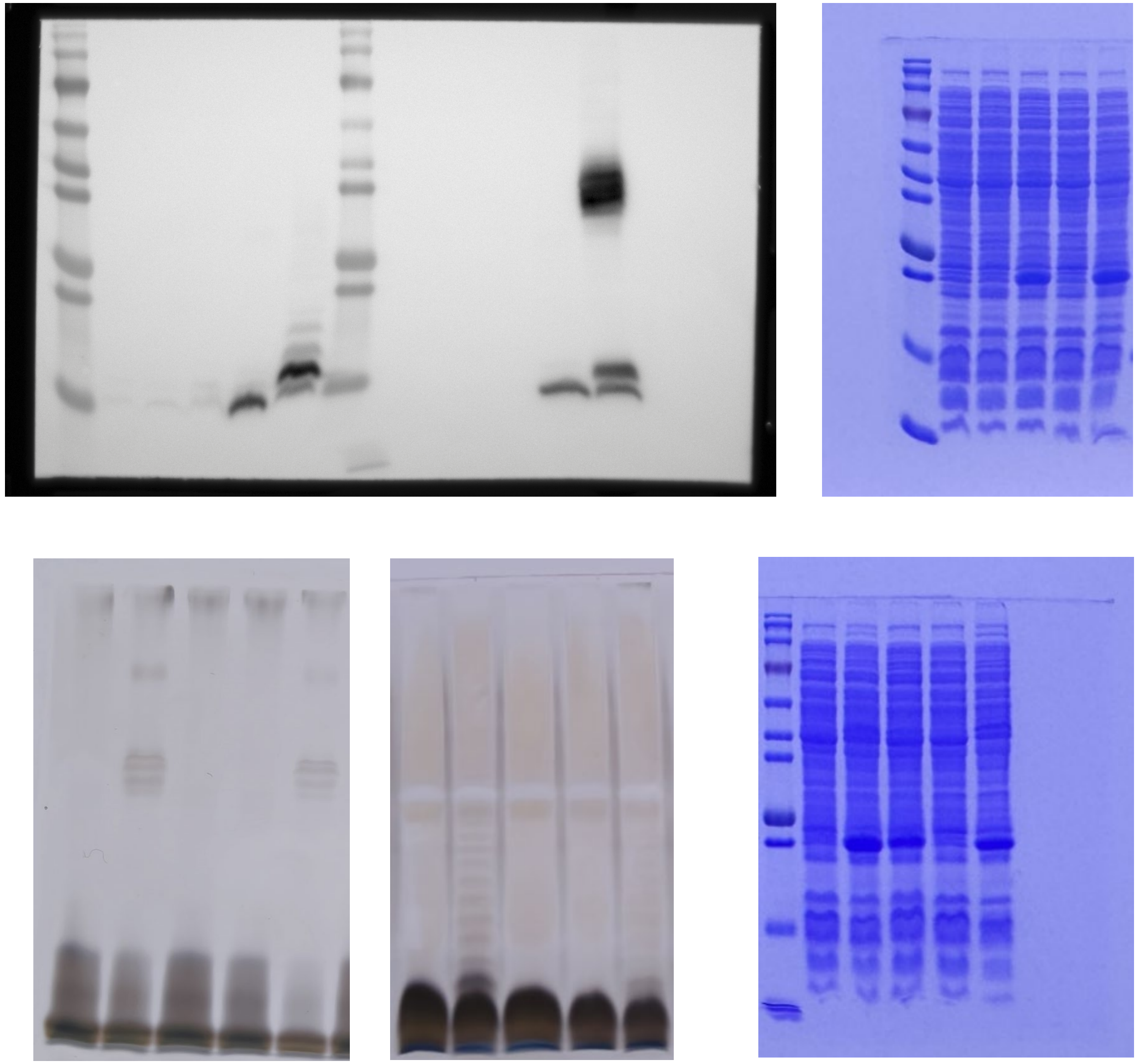

Figure 5b: Lane 1-3

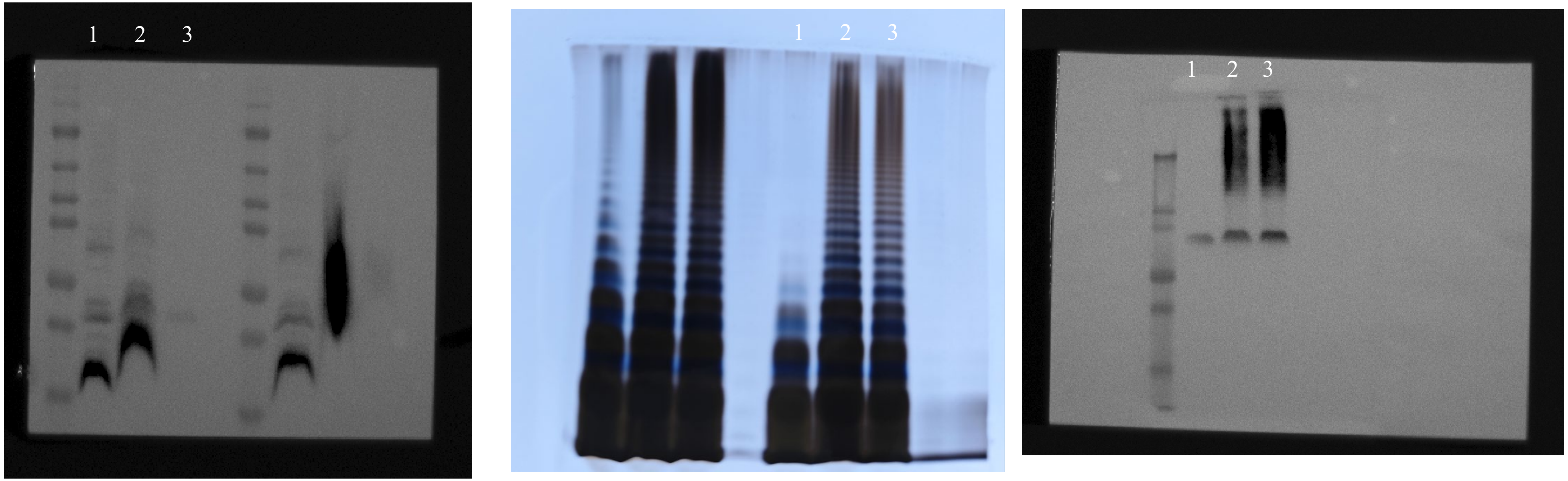

Figure S2: Lane 1-4

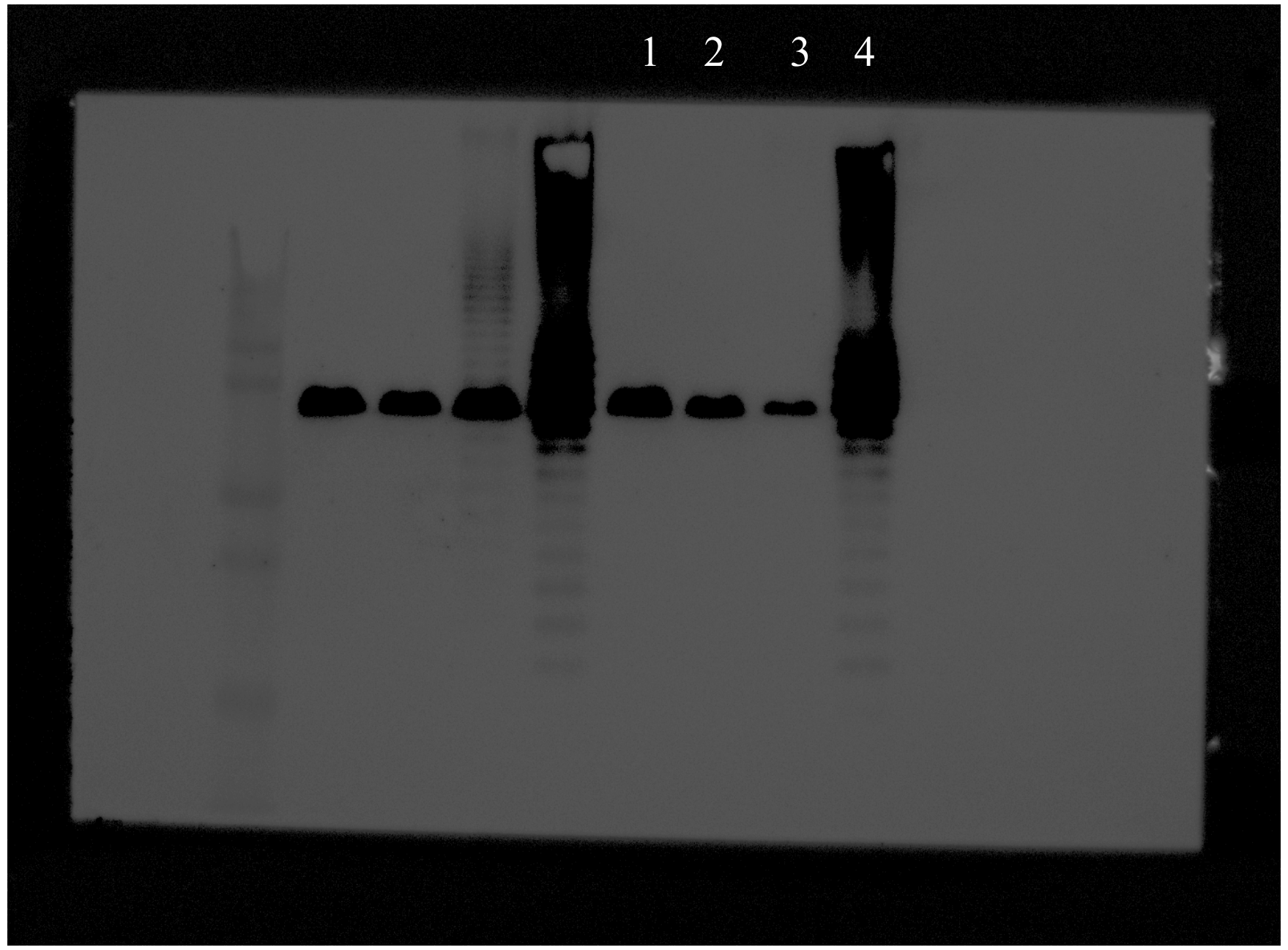

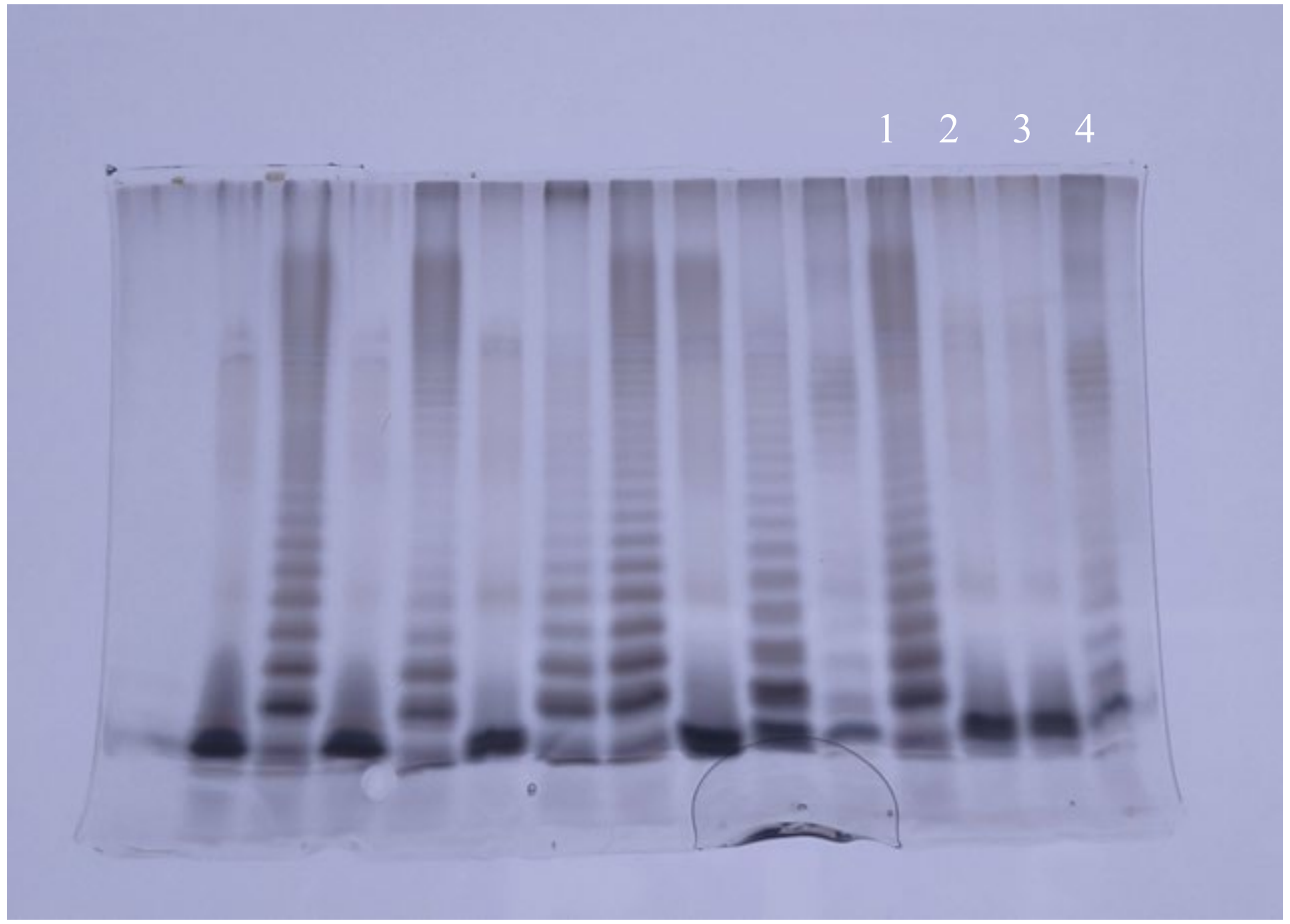

Figure S3: Lane 1-2

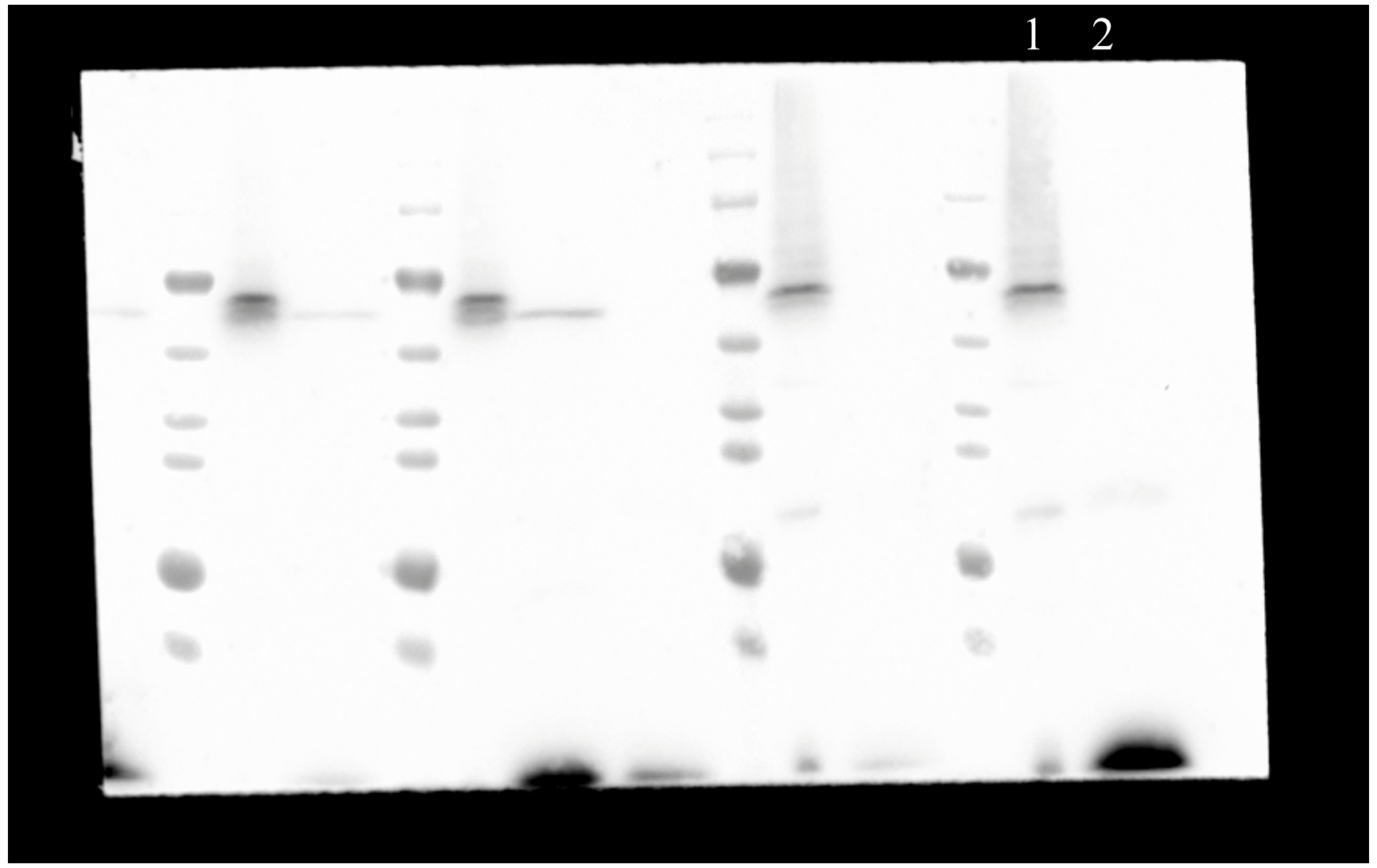

Figure S4a

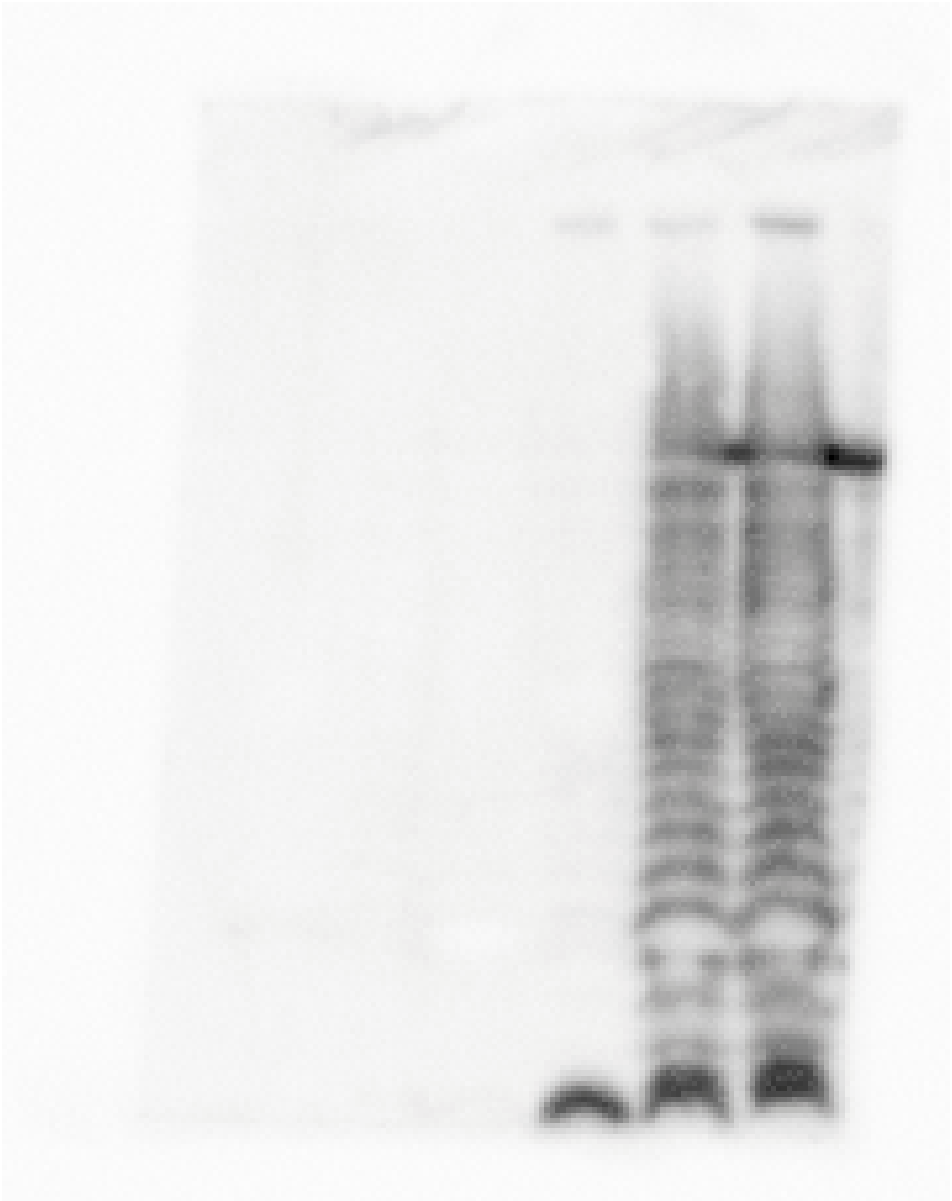

Figure S4b (right) Figure S5b (left)

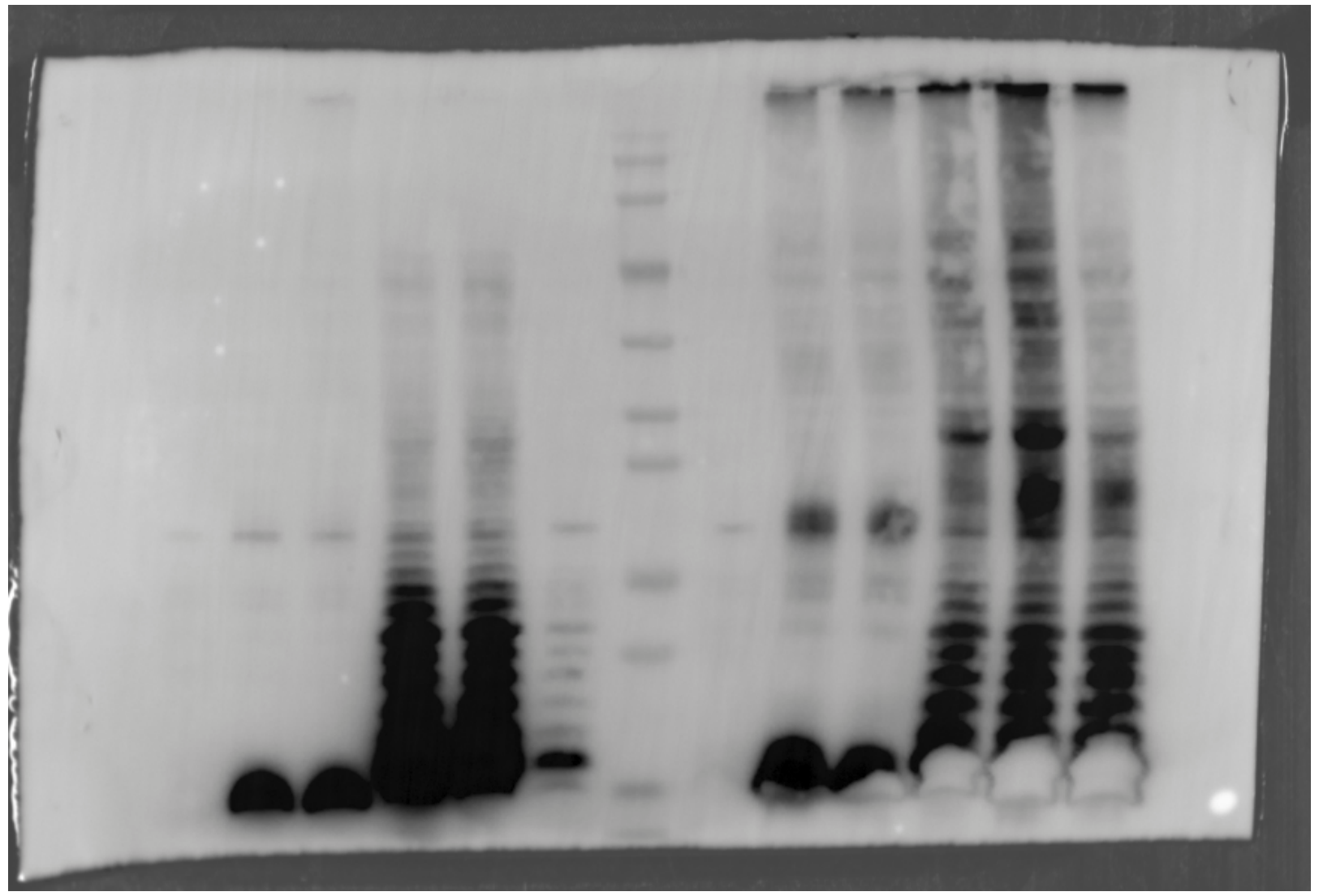

Figure S5a (right)

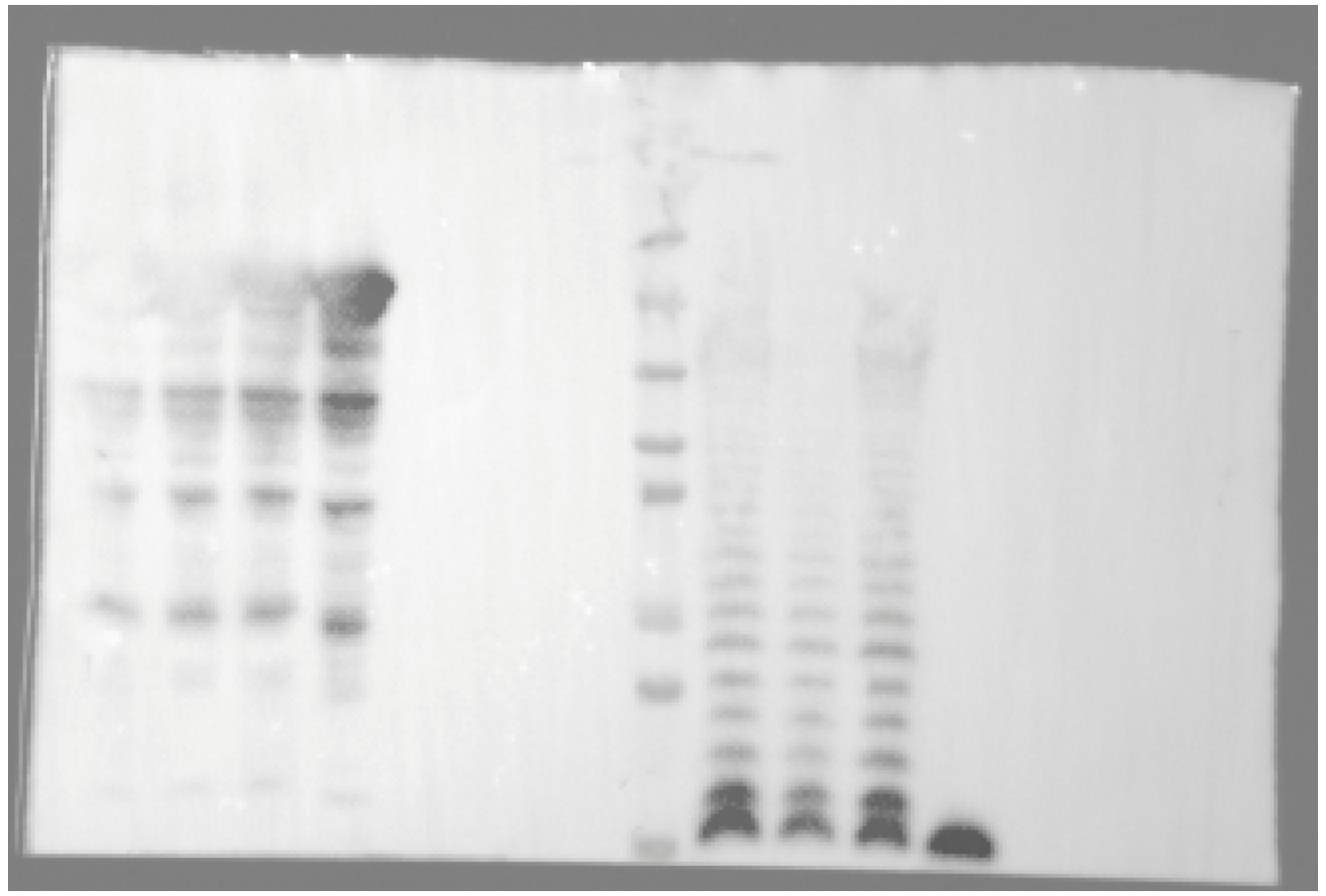

Figure S9a (right)

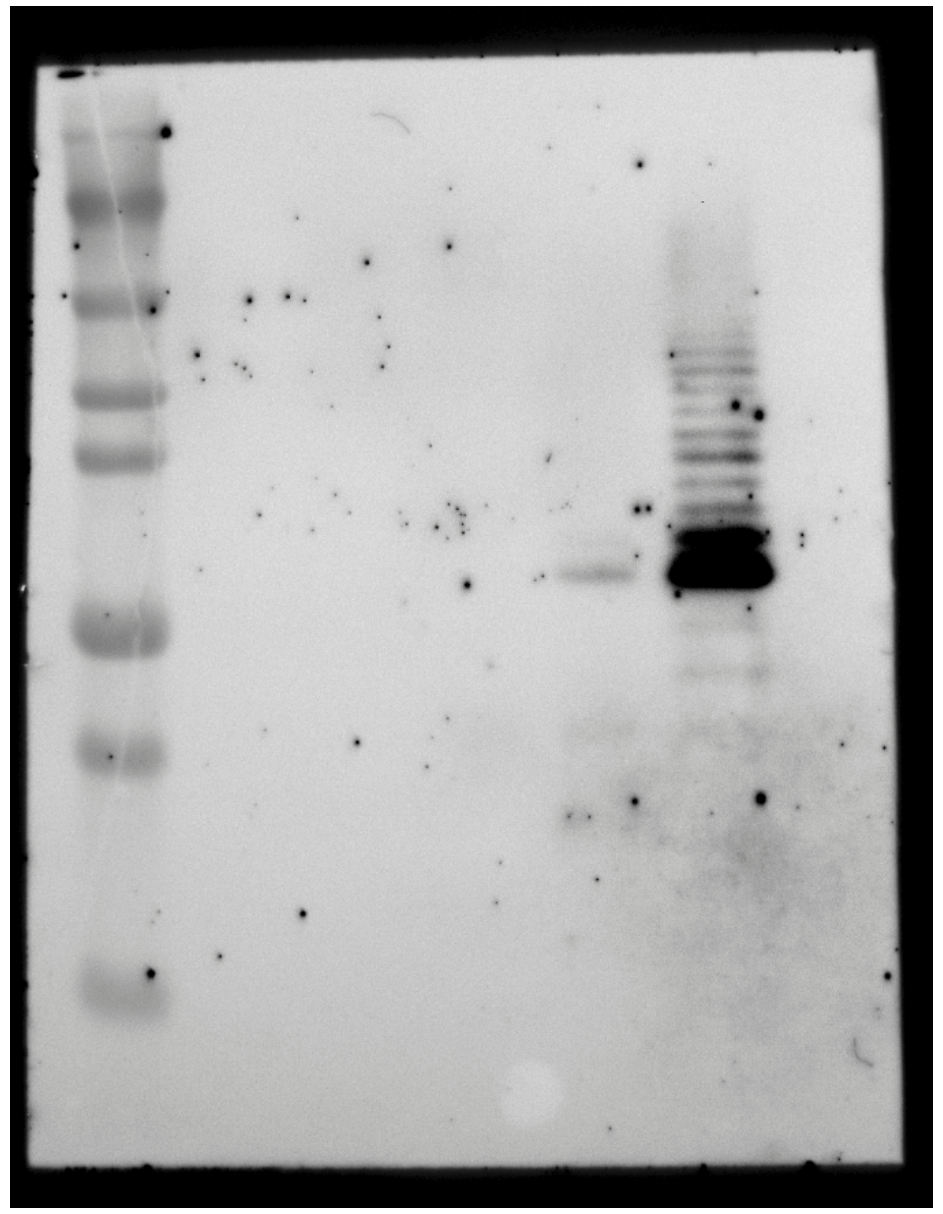

Figure S9b (left)

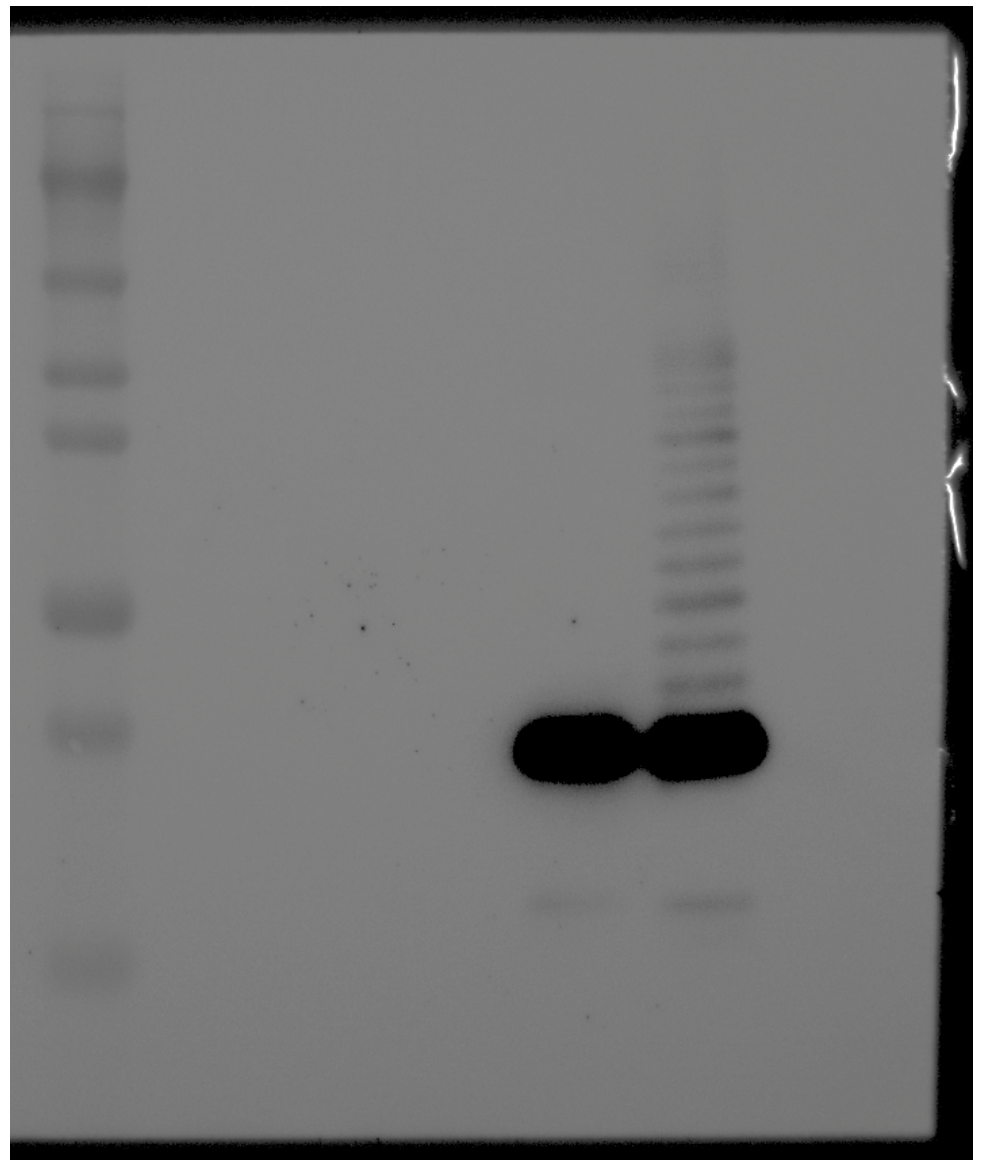

Supplement: Supplementary file 1 [file ppat.1014781.s015.pdf]
